# Supplementary figures and images for: Building synthetic biosensors using red blood cell proteins
Source: bioRxiv. 2023 Dec 16:2023.12.16.571988. Preprint. [Version 1] doi: 10.1101/2023.12.16.571988 (PMC10760168; doi:10.1101/2023.12.16.571988)

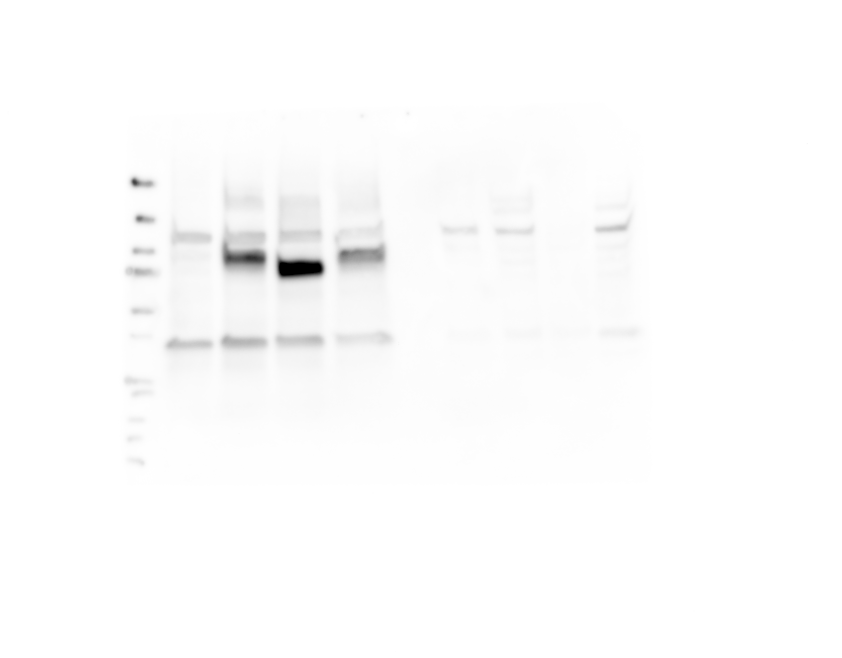

Supplement: Supplement 3 — • Supplementary Data 2: Raw and analyzed source data for Figures 2b, 2d, S3a–c, S4a–b, S6, 3b, 3d, S7c, s9e, 5c–f, S10–S12, 6c–d; source images for westerns blots (Figures s5b, s9b), and IVIS data (Figures 4b, 4d, 6, S8, S9e, S10, S12 (.Zip) [file media-3.zip › Supplementary Data 2/rawImages/S9b/S9b-Western-S12F5-0316-060654.tif]

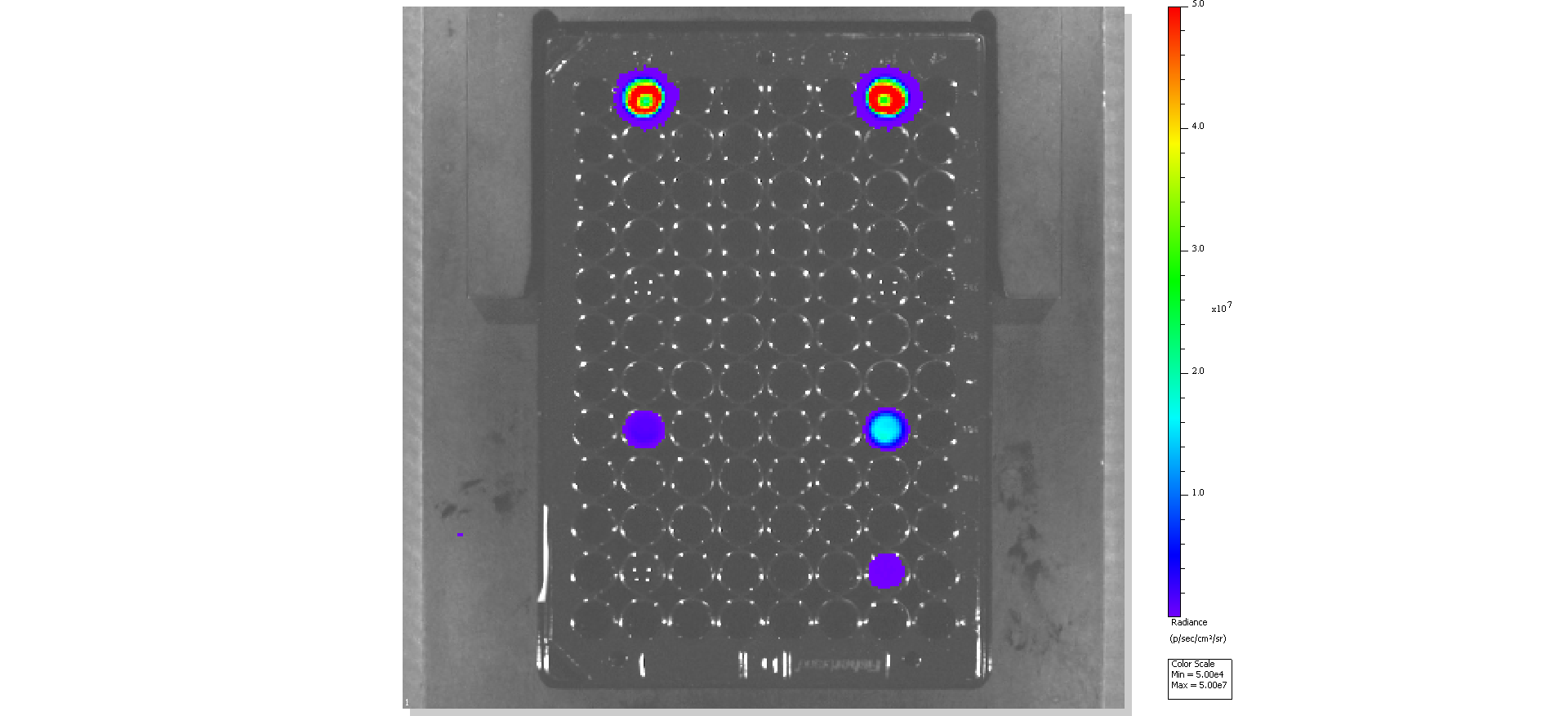

Supplement: Supplement 3 — • Supplementary Data 2: Raw and analyzed source data for Figures 2b, 2d, S3a–c, S4a–b, S6, 3b, 3d, S7c, s9e, 5c–f, S10–S12, 6c–d; source images for westerns blots (Figures s5b, s9b), and IVIS data (Figures 4b, 4d, 6, S8, S9e, S10, S12 (.Zip) [file media-3.zip › Supplementary Data 2/rawImages/S9e/S9e-0ham.png]

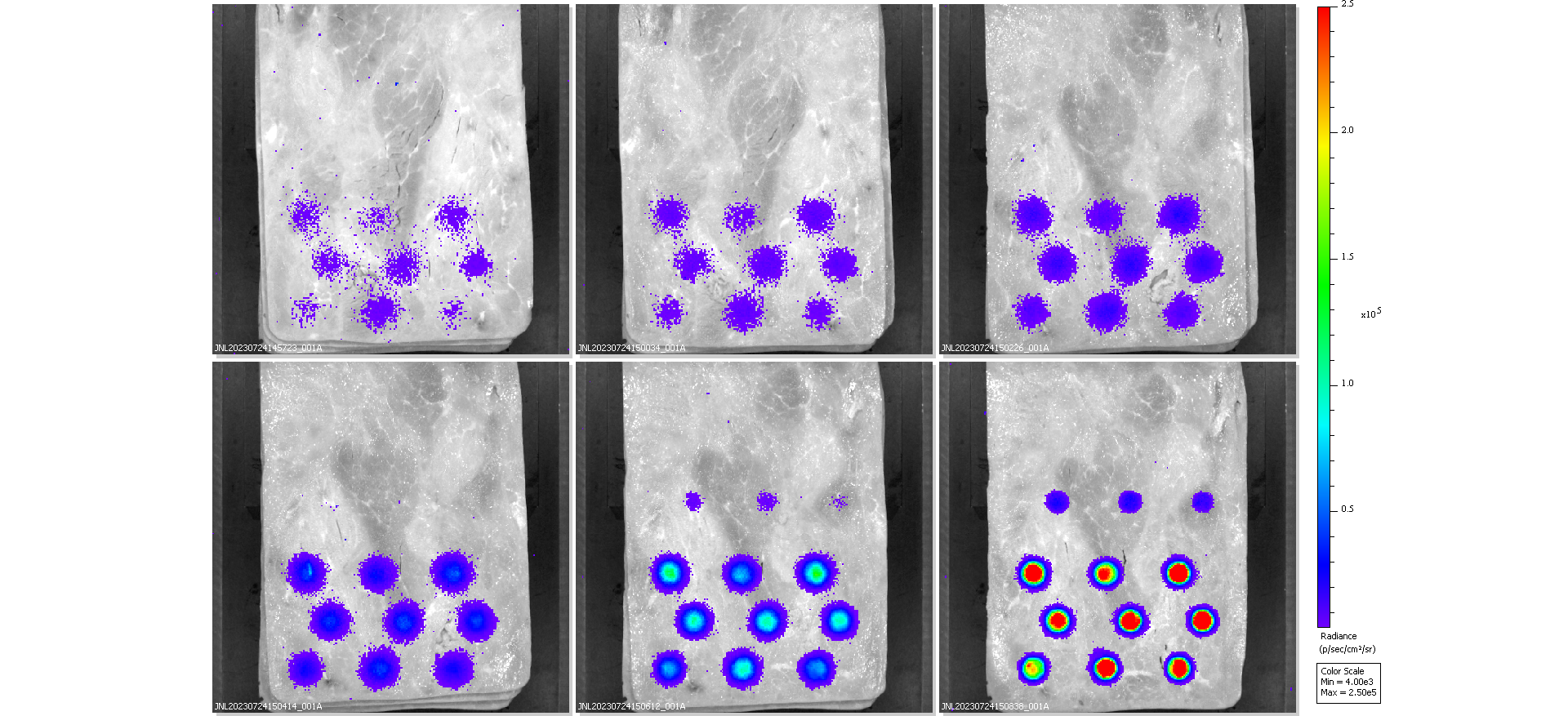

Supplement: Supplement 3 — • Supplementary Data 2: Raw and analyzed source data for Figures 2b, 2d, S3a–c, S4a–b, S6, 3b, 3d, S7c, s9e, 5c–f, S10–S12, 6c–d; source images for westerns blots (Figures s5b, s9b), and IVIS data (Figures 4b, 4d, 6, S8, S9e, S10, S12 (.Zip) [file media-3.zip › Supplementary Data 2/rawImages/s10/S10-allham.png]

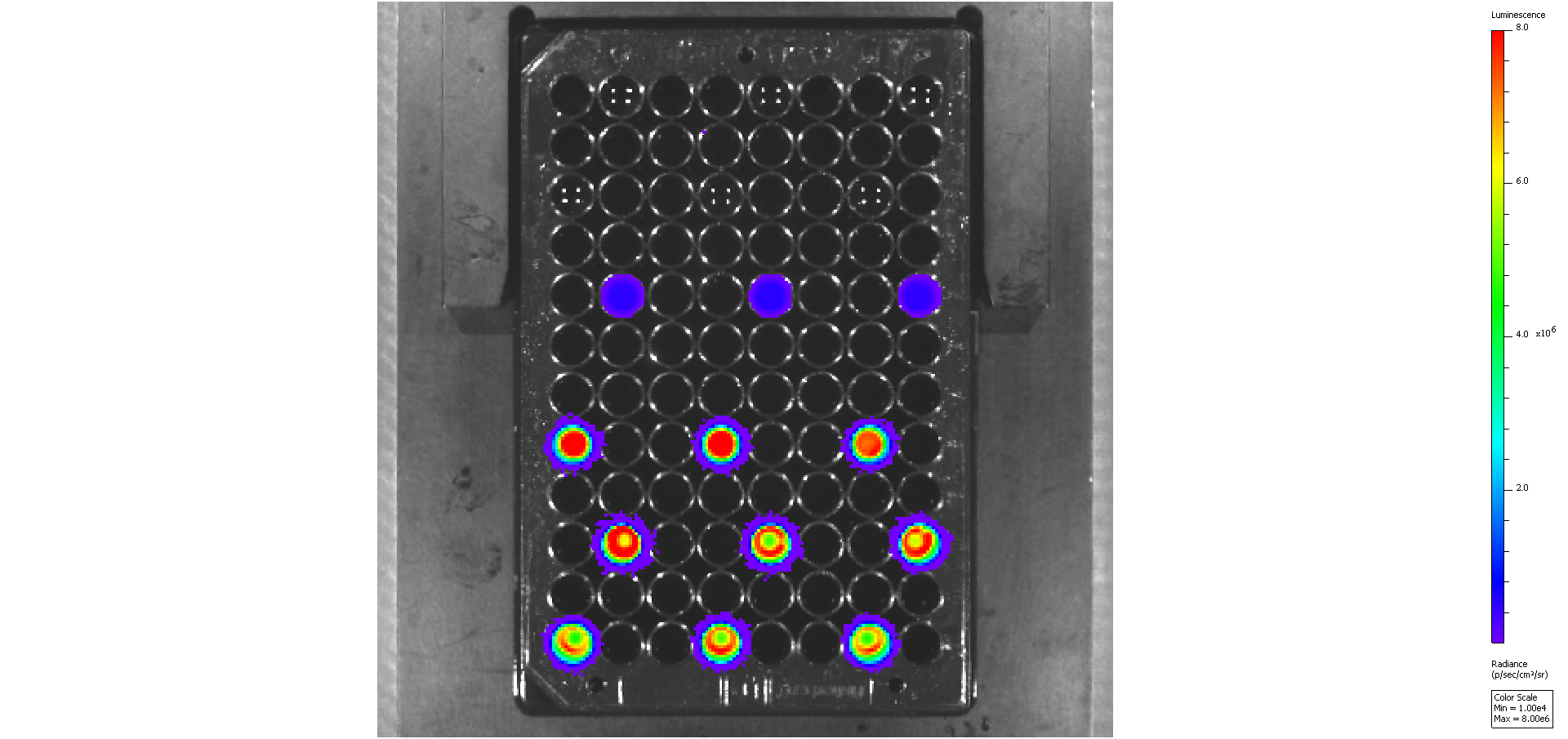

Supplement: Supplement 3 — • Supplementary Data 2: Raw and analyzed source data for Figures 2b, 2d, S3a–c, S4a–b, S6, 3b, 3d, S7c, s9e, 5c–f, S10–S12, 6c–d; source images for westerns blots (Figures s5b, s9b), and IVIS data (Figures 4b, 4d, 6, S8, S9e, S10, S12 (.Zip) [file media-3.zip › Supplementary Data 2/rawImages/s10/S10-0ham.png]

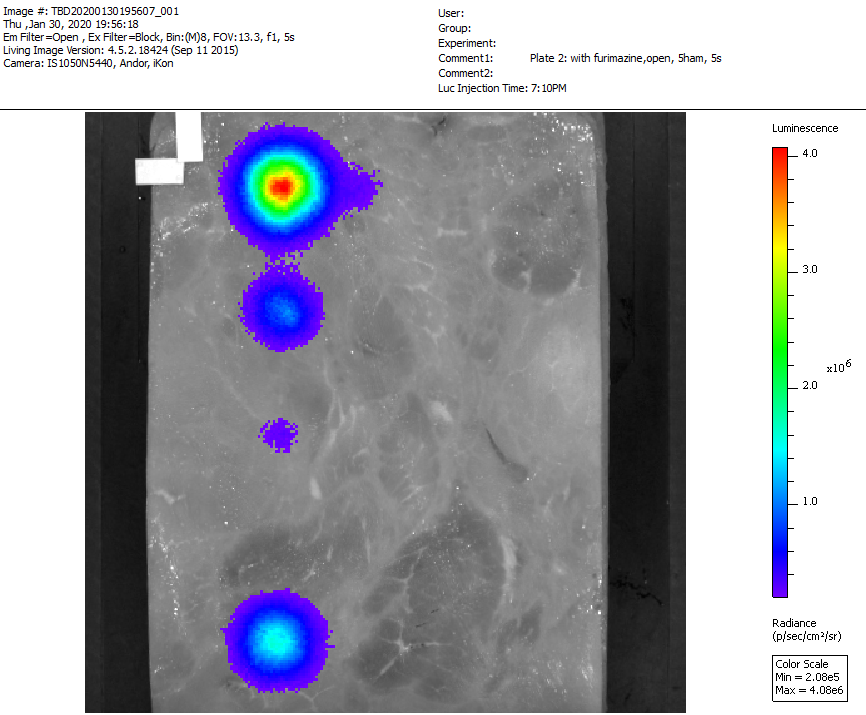

Supplement: Supplement 3 — • Supplementary Data 2: Raw and analyzed source data for Figures 2b, 2d, S3a–c, S4a–b, S6, 3b, 3d, S7c, s9e, 5c–f, S10–S12, 6c–d; source images for westerns blots (Figures s5b, s9b), and IVIS data (Figures 4b, 4d, 6, S8, S9e, S10, S12 (.Zip) [file media-3.zip › Supplementary Data 2/rawImages/S8a_4b/2-5s-5ham.png]

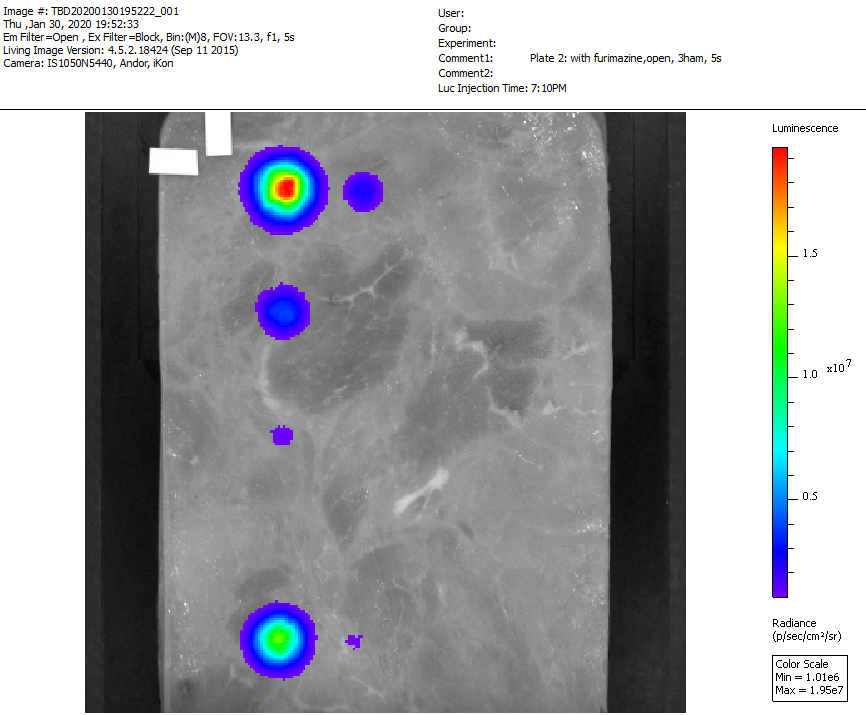

Supplement: Supplement 3 — • Supplementary Data 2: Raw and analyzed source data for Figures 2b, 2d, S3a–c, S4a–b, S6, 3b, 3d, S7c, s9e, 5c–f, S10–S12, 6c–d; source images for westerns blots (Figures s5b, s9b), and IVIS data (Figures 4b, 4d, 6, S8, S9e, S10, S12 (.Zip) [file media-3.zip › Supplementary Data 2/rawImages/S8a_4b/2-5s-3ham.png]

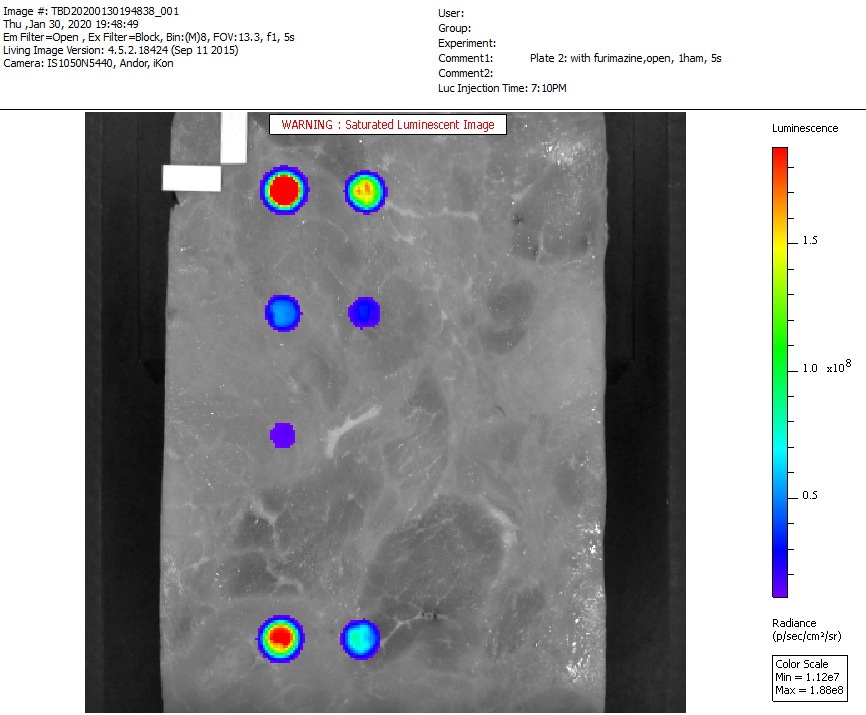

Supplement: Supplement 3 — • Supplementary Data 2: Raw and analyzed source data for Figures 2b, 2d, S3a–c, S4a–b, S6, 3b, 3d, S7c, s9e, 5c–f, S10–S12, 6c–d; source images for westerns blots (Figures s5b, s9b), and IVIS data (Figures 4b, 4d, 6, S8, S9e, S10, S12 (.Zip) [file media-3.zip › Supplementary Data 2/rawImages/S8a_4b/2-5s-1ham.png]

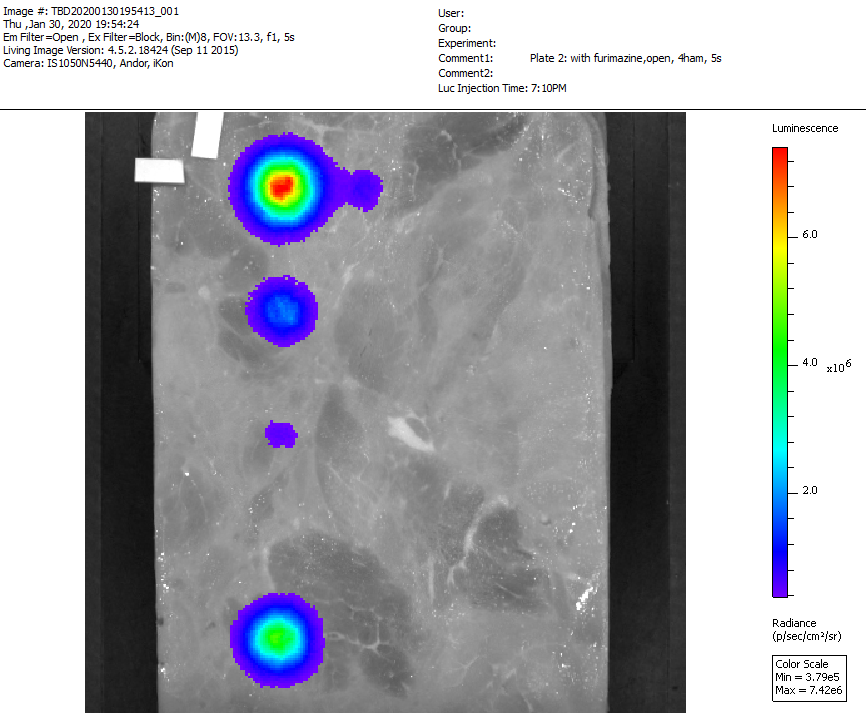

Supplement: Supplement 3 — • Supplementary Data 2: Raw and analyzed source data for Figures 2b, 2d, S3a–c, S4a–b, S6, 3b, 3d, S7c, s9e, 5c–f, S10–S12, 6c–d; source images for westerns blots (Figures s5b, s9b), and IVIS data (Figures 4b, 4d, 6, S8, S9e, S10, S12 (.Zip) [file media-3.zip › Supplementary Data 2/rawImages/S8a_4b/2-5s-4ham.png]

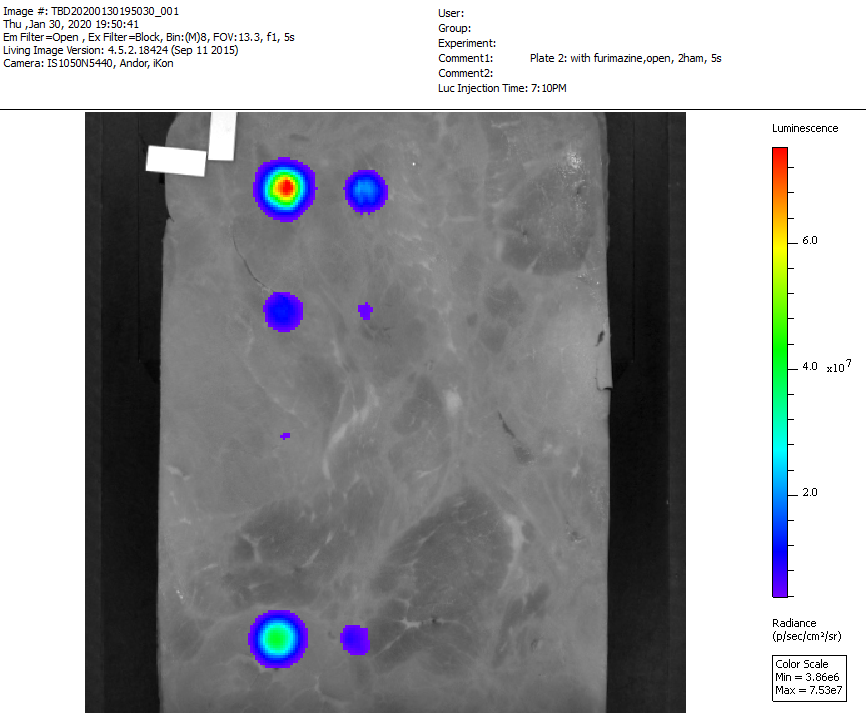

Supplement: Supplement 3 — • Supplementary Data 2: Raw and analyzed source data for Figures 2b, 2d, S3a–c, S4a–b, S6, 3b, 3d, S7c, s9e, 5c–f, S10–S12, 6c–d; source images for westerns blots (Figures s5b, s9b), and IVIS data (Figures 4b, 4d, 6, S8, S9e, S10, S12 (.Zip) [file media-3.zip › Supplementary Data 2/rawImages/S8a_4b/2-5s-2ham.png]

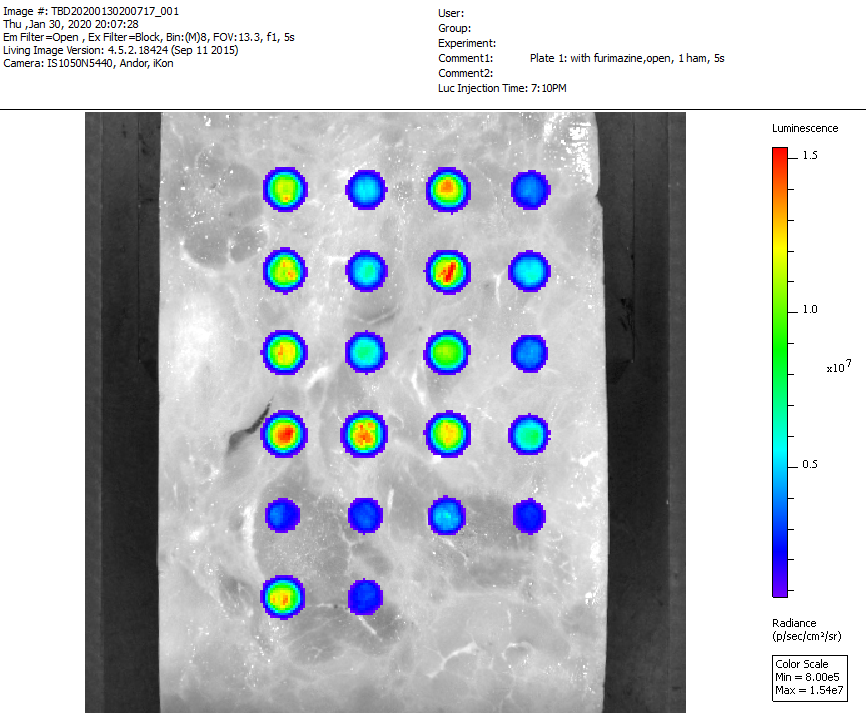

Supplement: Supplement 3 — • Supplementary Data 2: Raw and analyzed source data for Figures 2b, 2d, S3a–c, S4a–b, S6, 3b, 3d, S7c, s9e, 5c–f, S10–S12, 6c–d; source images for westerns blots (Figures s5b, s9b), and IVIS data (Figures 4b, 4d, 6, S8, S9e, S10, S12 (.Zip) [file media-3.zip › Supplementary Data 2/rawImages/S8b_4d/1-5s-1ham.png]

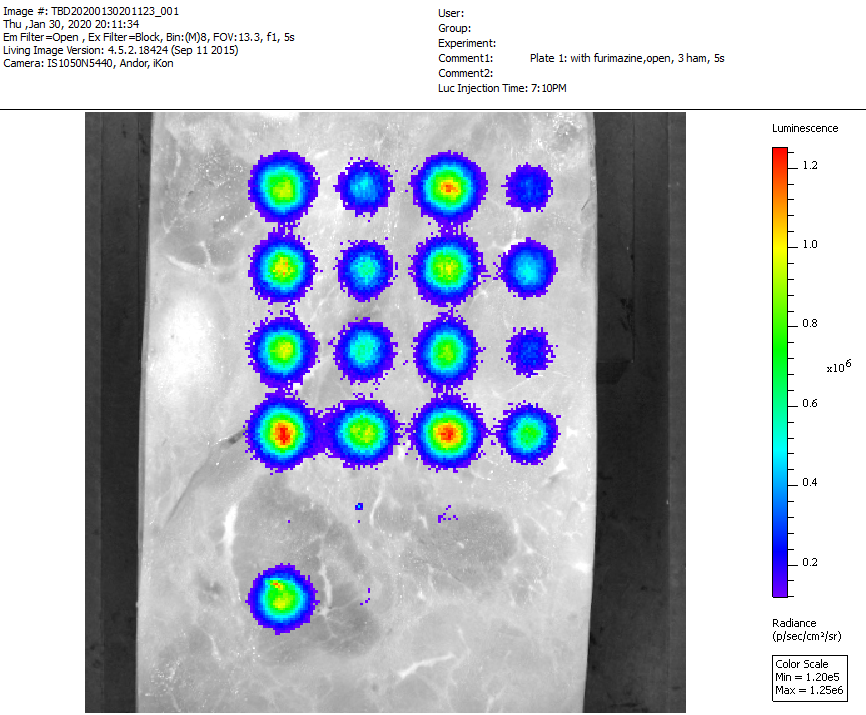

Supplement: Supplement 3 — • Supplementary Data 2: Raw and analyzed source data for Figures 2b, 2d, S3a–c, S4a–b, S6, 3b, 3d, S7c, s9e, 5c–f, S10–S12, 6c–d; source images for westerns blots (Figures s5b, s9b), and IVIS data (Figures 4b, 4d, 6, S8, S9e, S10, S12 (.Zip) [file media-3.zip › Supplementary Data 2/rawImages/S8b_4d/1-5s-3ham.png]

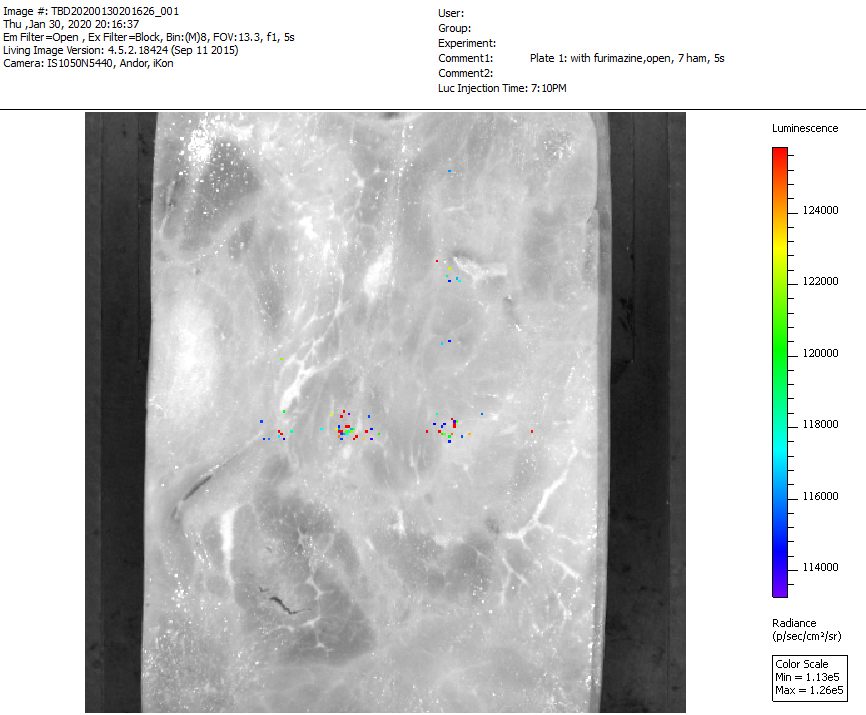

Supplement: Supplement 3 — • Supplementary Data 2: Raw and analyzed source data for Figures 2b, 2d, S3a–c, S4a–b, S6, 3b, 3d, S7c, s9e, 5c–f, S10–S12, 6c–d; source images for westerns blots (Figures s5b, s9b), and IVIS data (Figures 4b, 4d, 6, S8, S9e, S10, S12 (.Zip) [file media-3.zip › Supplementary Data 2/rawImages/S8b_4d/1-5s-7ham.png]

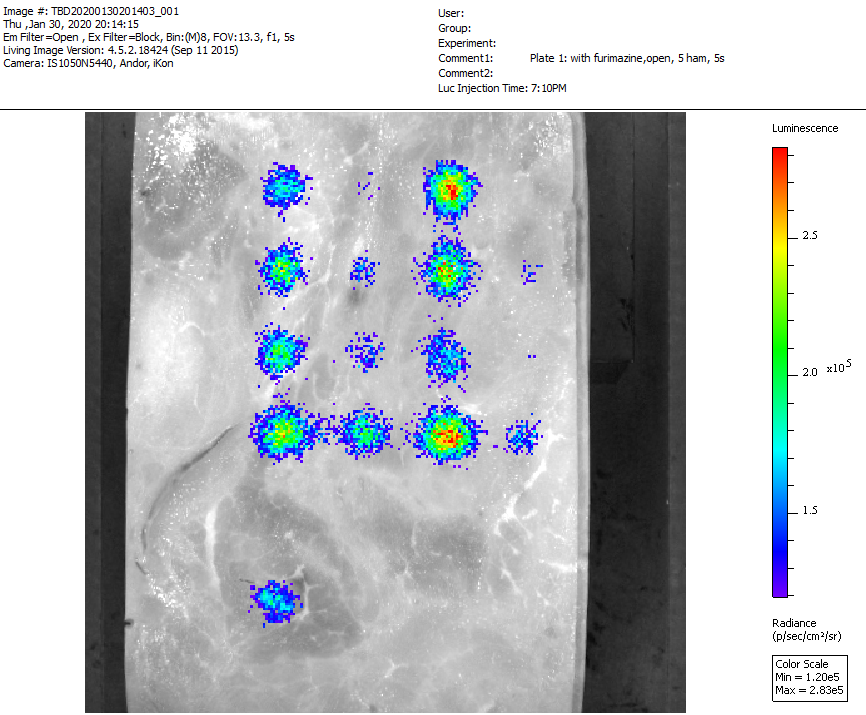

Supplement: Supplement 3 — • Supplementary Data 2: Raw and analyzed source data for Figures 2b, 2d, S3a–c, S4a–b, S6, 3b, 3d, S7c, s9e, 5c–f, S10–S12, 6c–d; source images for westerns blots (Figures s5b, s9b), and IVIS data (Figures 4b, 4d, 6, S8, S9e, S10, S12 (.Zip) [file media-3.zip › Supplementary Data 2/rawImages/S8b_4d/1-5s-5ham.png]

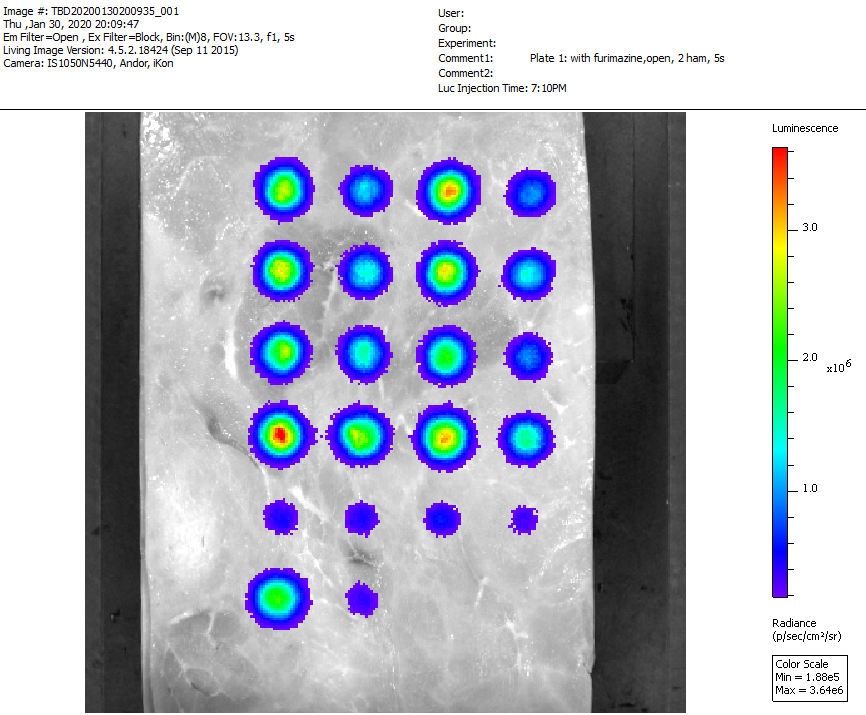

Supplement: Supplement 3 — • Supplementary Data 2: Raw and analyzed source data for Figures 2b, 2d, S3a–c, S4a–b, S6, 3b, 3d, S7c, s9e, 5c–f, S10–S12, 6c–d; source images for westerns blots (Figures s5b, s9b), and IVIS data (Figures 4b, 4d, 6, S8, S9e, S10, S12 (.Zip) [file media-3.zip › Supplementary Data 2/rawImages/S8b_4d/1-5s-2ham.png]

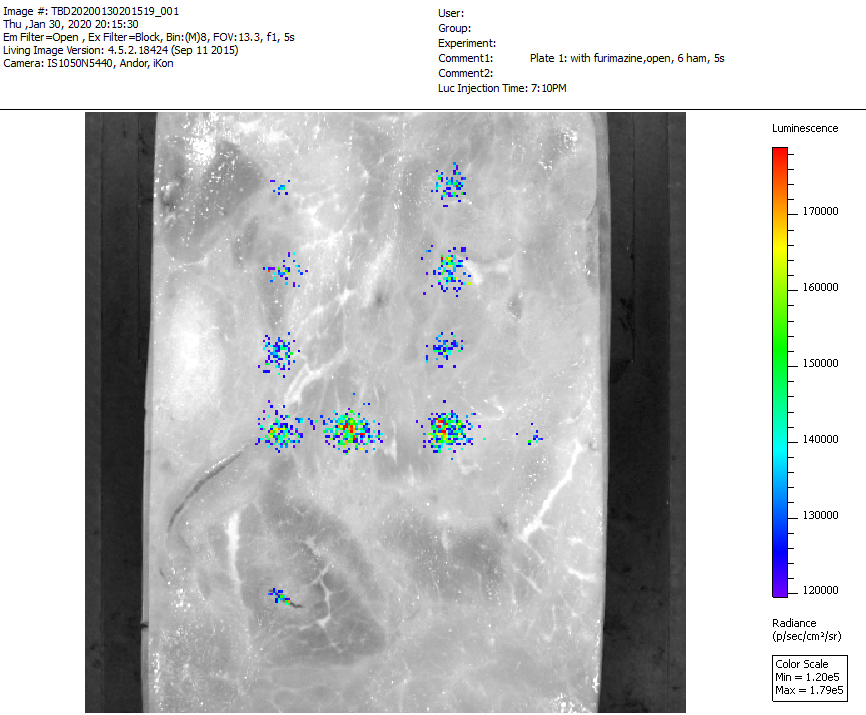

Supplement: Supplement 3 — • Supplementary Data 2: Raw and analyzed source data for Figures 2b, 2d, S3a–c, S4a–b, S6, 3b, 3d, S7c, s9e, 5c–f, S10–S12, 6c–d; source images for westerns blots (Figures s5b, s9b), and IVIS data (Figures 4b, 4d, 6, S8, S9e, S10, S12 (.Zip) [file media-3.zip › Supplementary Data 2/rawImages/S8b_4d/1-5s-6ham.png]

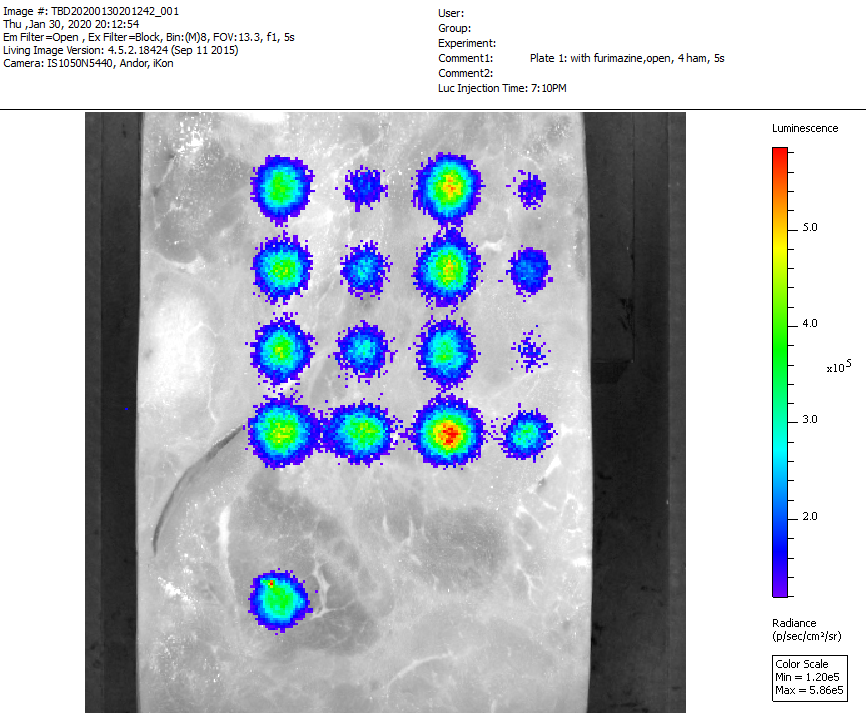

Supplement: Supplement 3 — • Supplementary Data 2: Raw and analyzed source data for Figures 2b, 2d, S3a–c, S4a–b, S6, 3b, 3d, S7c, s9e, 5c–f, S10–S12, 6c–d; source images for westerns blots (Figures s5b, s9b), and IVIS data (Figures 4b, 4d, 6, S8, S9e, S10, S12 (.Zip) [file media-3.zip › Supplementary Data 2/rawImages/S8b_4d/1-5s-4ham.png]

3/15 1min

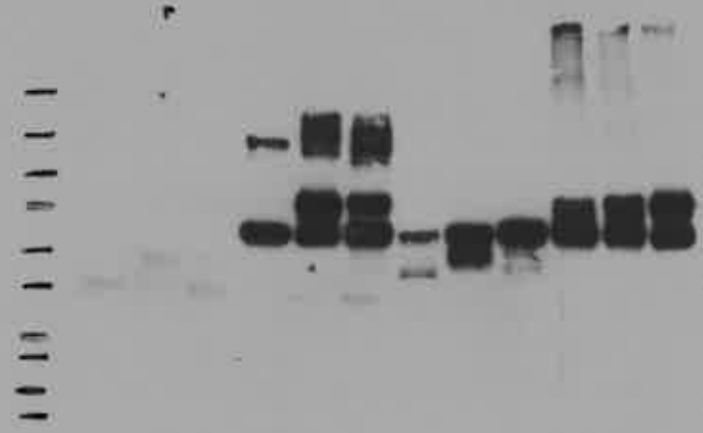

3/15 2min

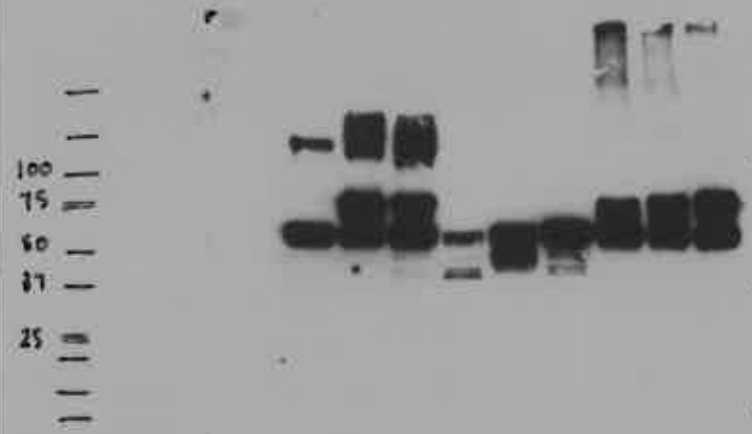

Supplement: Supplement 3 — • Supplementary Data 2: Raw and analyzed source data for Figures 2b, 2d, S3a–c, S4a–b, S6, 3b, 3d, S7c, s9e, 5c–f, S10–S12, 6c–d; source images for westerns blots (Figures s5b, s9b), and IVIS data (Figures 4b, 4d, 6, S8, S9e, S10, S12 (.Zip) [file media-3.zip › Supplementary Data 2/rawImages/S5b/S5b-Western 3.15.16 b&w.pdf]

3/15 2-100

100  
75  
50  
25

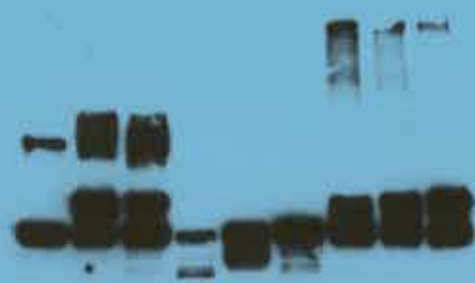

3/15 1-100

100  
75  
50  
25

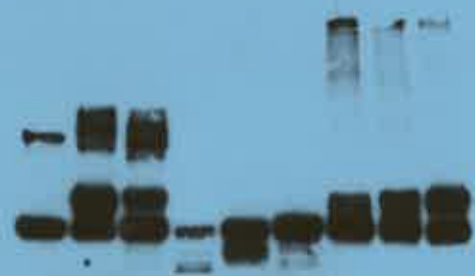

Supplement: Supplement 3 — • Supplementary Data 2: Raw and analyzed source data for Figures 2b, 2d, S3a–c, S4a–b, S6, 3b, 3d, S7c, s9e, 5c–f, S10–S12, 6c–d; source images for westerns blots (Figures s5b, s9b), and IVIS data (Figures 4b, 4d, 6, S8, S9e, S10, S12 (.Zip) [file media-3.zip › Supplementary Data 2/rawImages/S5b/S5b-Western 3.15.16 color.pdf]

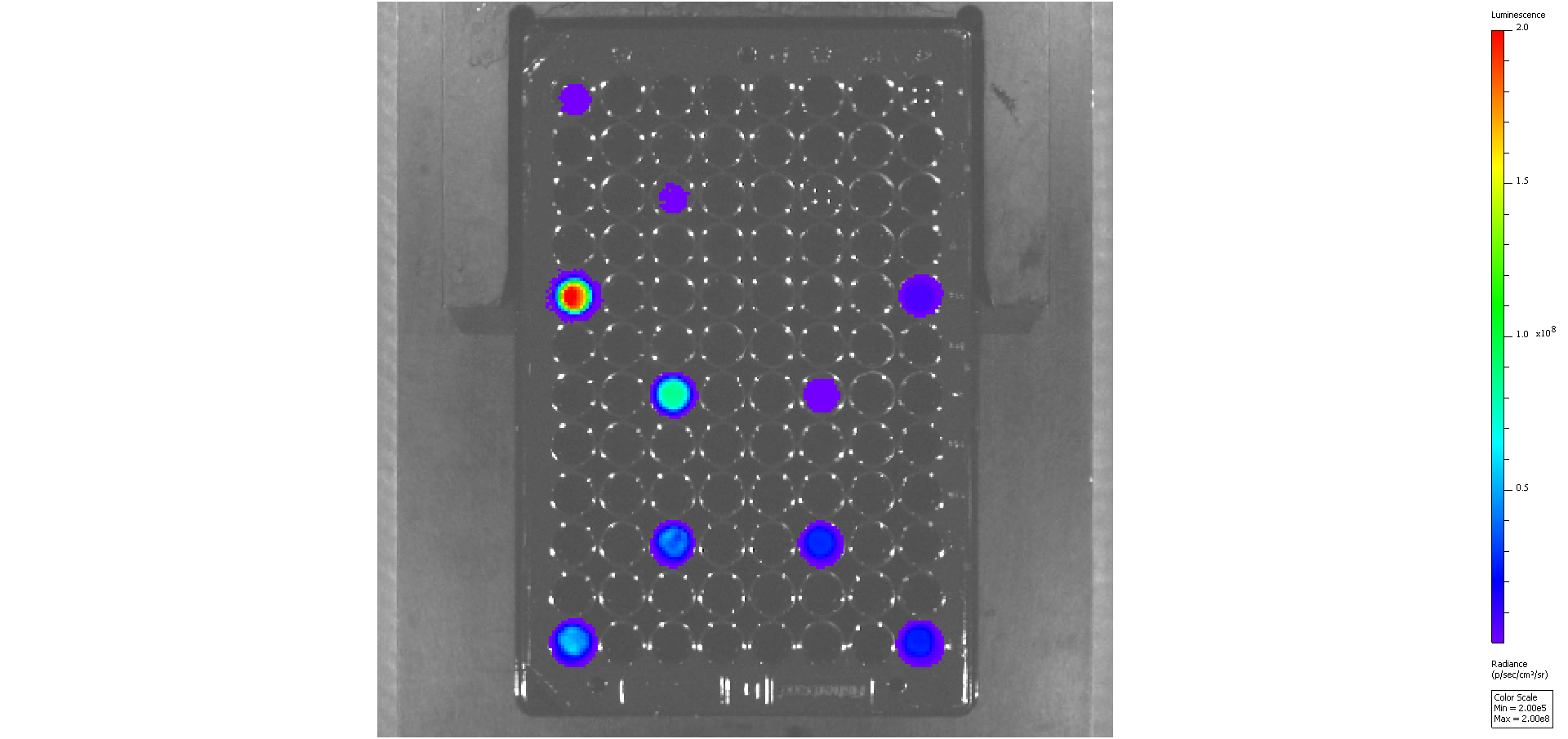

Supplement: Supplement 3 — • Supplementary Data 2: Raw and analyzed source data for Figures 2b, 2d, S3a–c, S4a–b, S6, 3b, 3d, S7c, s9e, 5c–f, S10–S12, 6c–d; source images for westerns blots (Figures s5b, s9b), and IVIS data (Figures 4b, 4d, 6, S8, S9e, S10, S12 (.Zip) [file media-3.zip › Supplementary Data 2/rawImages/s12/s12-rep3-0ham.png]

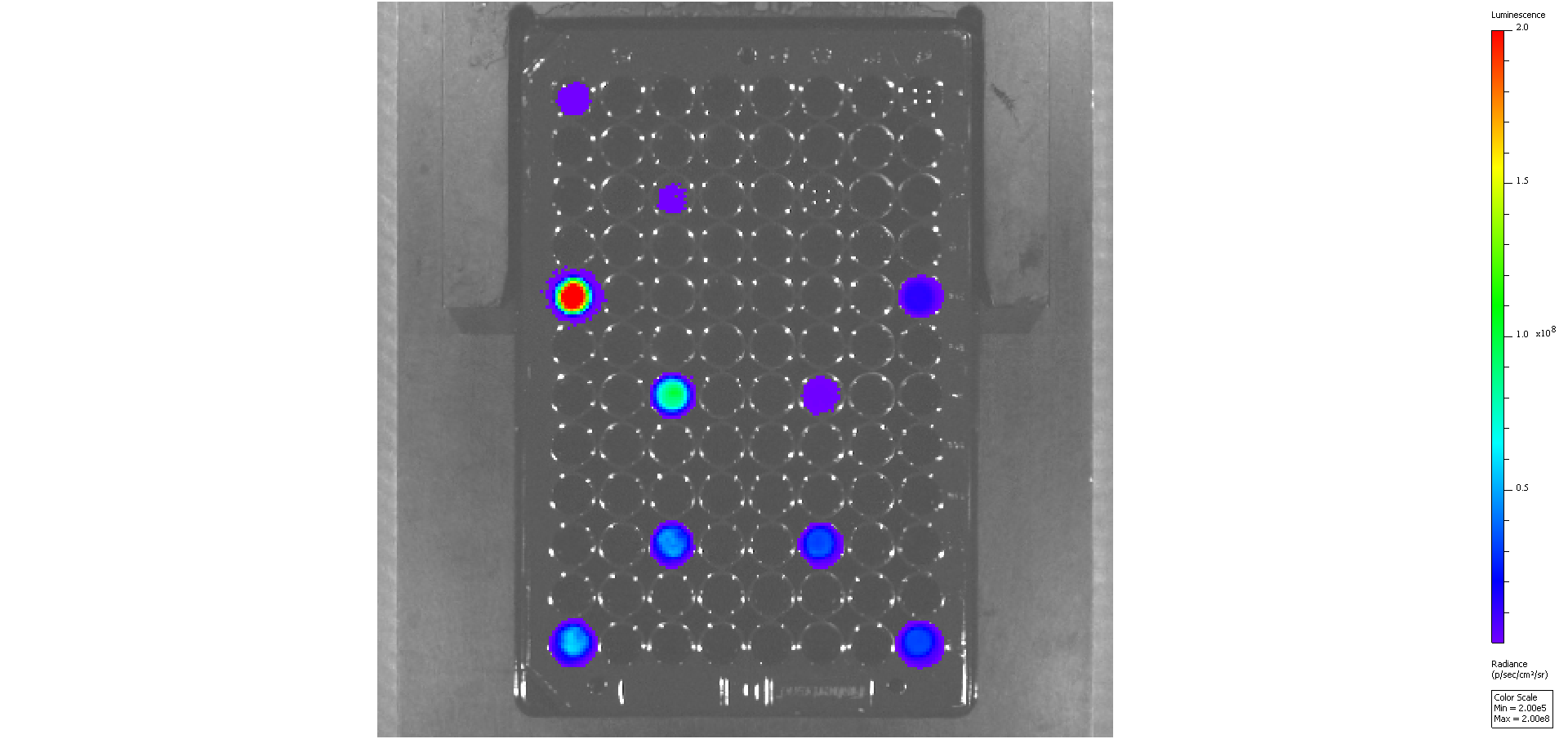

Supplement: Supplement 3 — • Supplementary Data 2: Raw and analyzed source data for Figures 2b, 2d, S3a–c, S4a–b, S6, 3b, 3d, S7c, s9e, 5c–f, S10–S12, 6c–d; source images for westerns blots (Figures s5b, s9b), and IVIS data (Figures 4b, 4d, 6, S8, S9e, S10, S12 (.Zip) [file media-3.zip › Supplementary Data 2/rawImages/s12/s12-rep2-0ham.png]

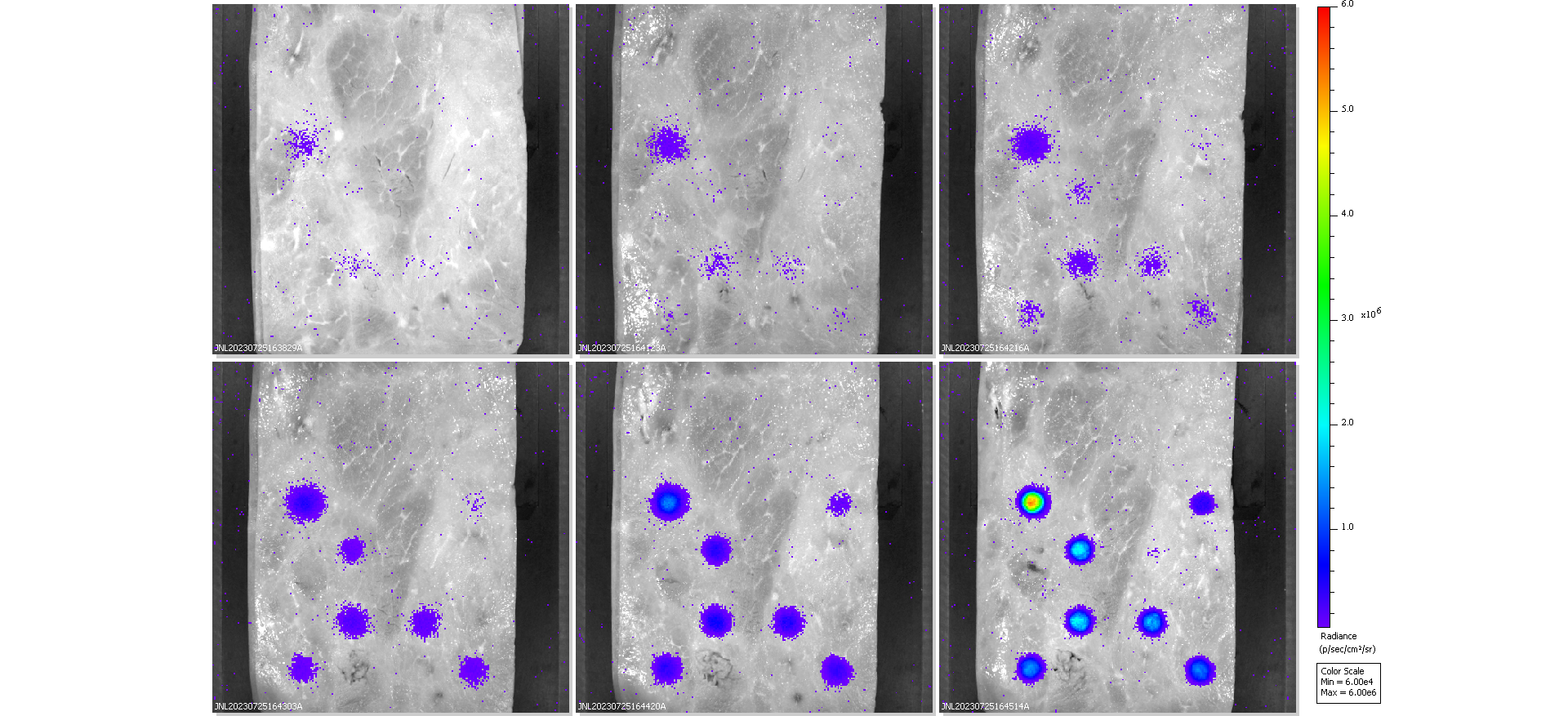

Supplement: Supplement 3 — • Supplementary Data 2: Raw and analyzed source data for Figures 2b, 2d, S3a–c, S4a–b, S6, 3b, 3d, S7c, s9e, 5c–f, S10–S12, 6c–d; source images for westerns blots (Figures s5b, s9b), and IVIS data (Figures 4b, 4d, 6, S8, S9e, S10, S12 (.Zip) [file media-3.zip › Supplementary Data 2/rawImages/s12/s12-rep2-allham.png]

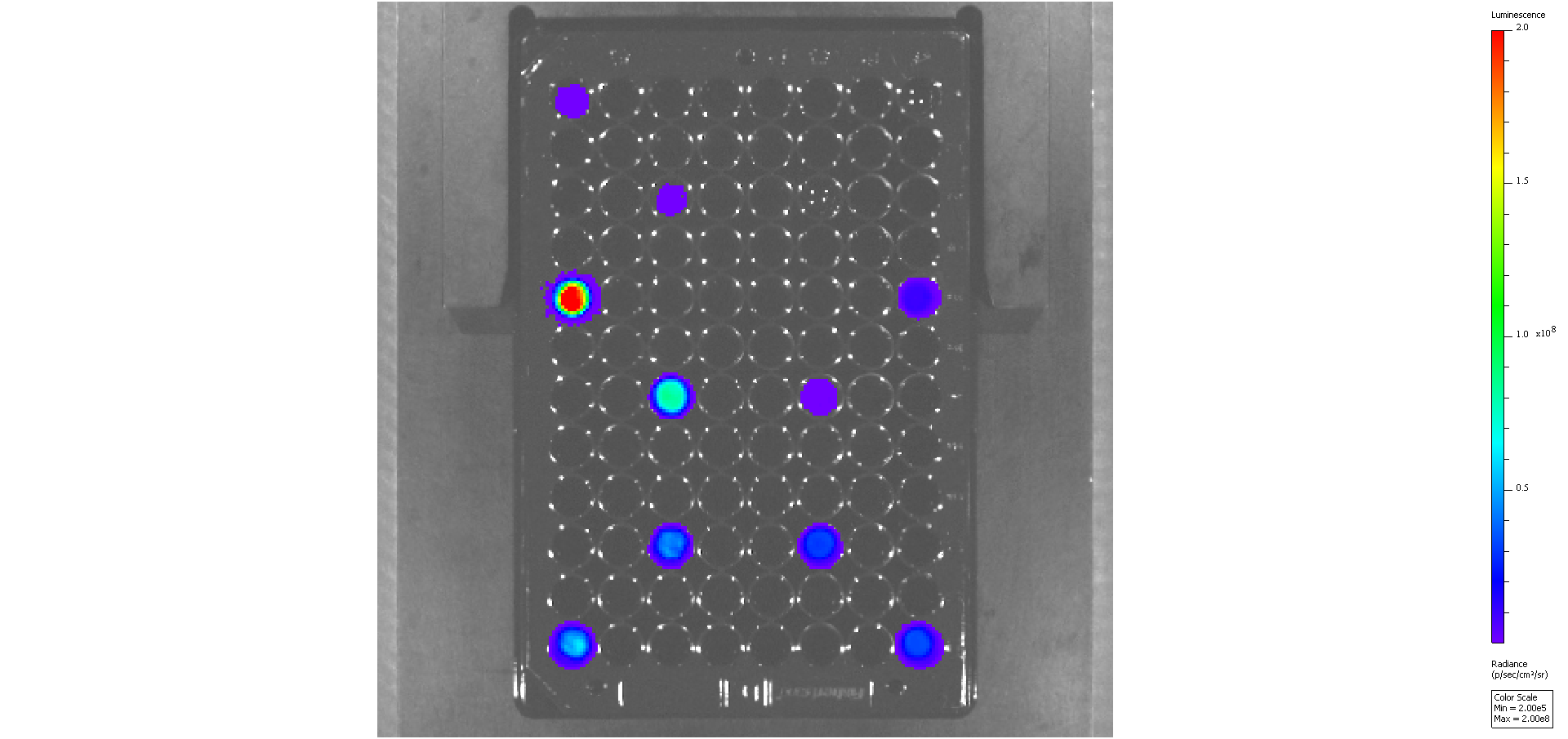

Supplement: Supplement 3 — • Supplementary Data 2: Raw and analyzed source data for Figures 2b, 2d, S3a–c, S4a–b, S6, 3b, 3d, S7c, s9e, 5c–f, S10–S12, 6c–d; source images for westerns blots (Figures s5b, s9b), and IVIS data (Figures 4b, 4d, 6, S8, S9e, S10, S12 (.Zip) [file media-3.zip › Supplementary Data 2/rawImages/s12/s12-rep1-0ham.png]

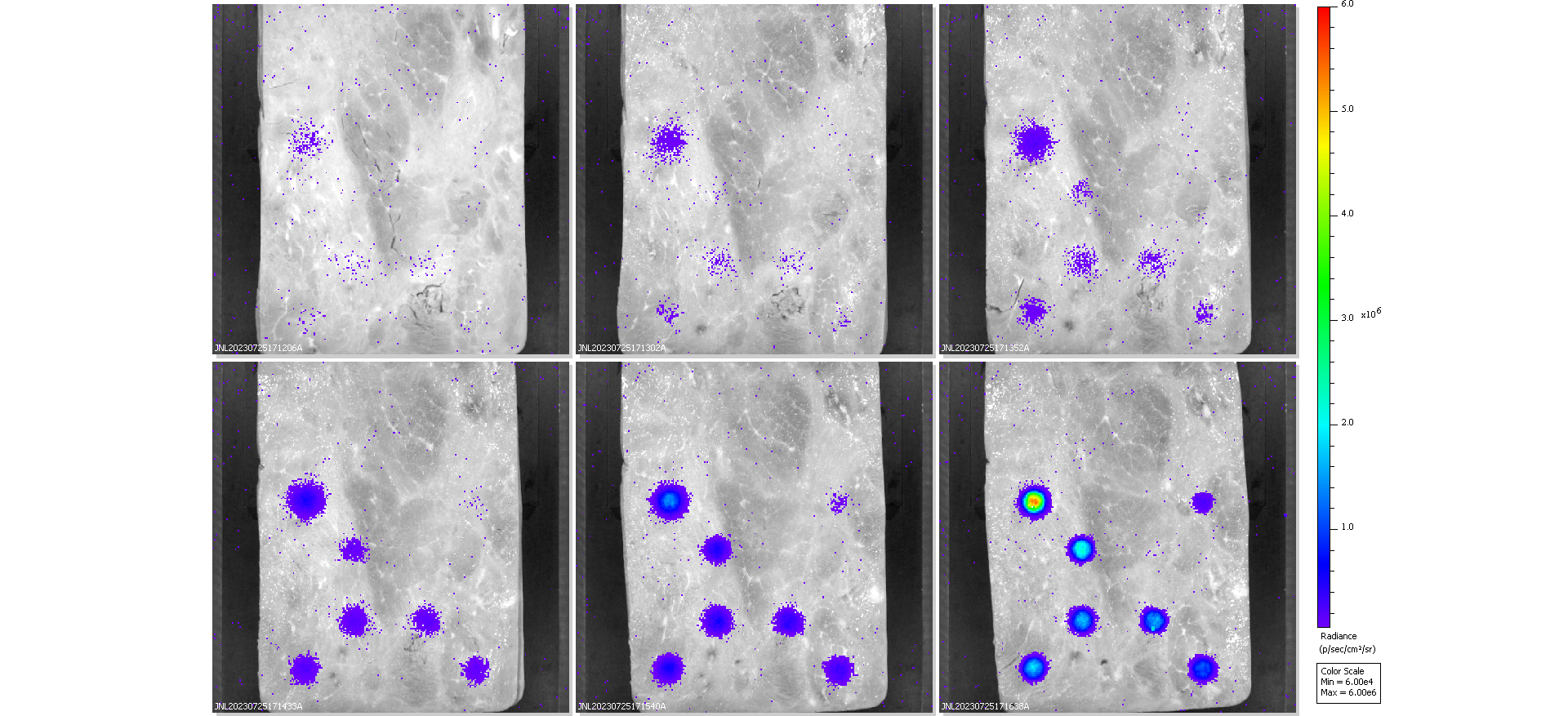

Supplement: Supplement 3 — • Supplementary Data 2: Raw and analyzed source data for Figures 2b, 2d, S3a–c, S4a–b, S6, 3b, 3d, S7c, s9e, 5c–f, S10–S12, 6c–d; source images for westerns blots (Figures s5b, s9b), and IVIS data (Figures 4b, 4d, 6, S8, S9e, S10, S12 (.Zip) [file media-3.zip › Supplementary Data 2/rawImages/s12/s12-rep3-allham.png]

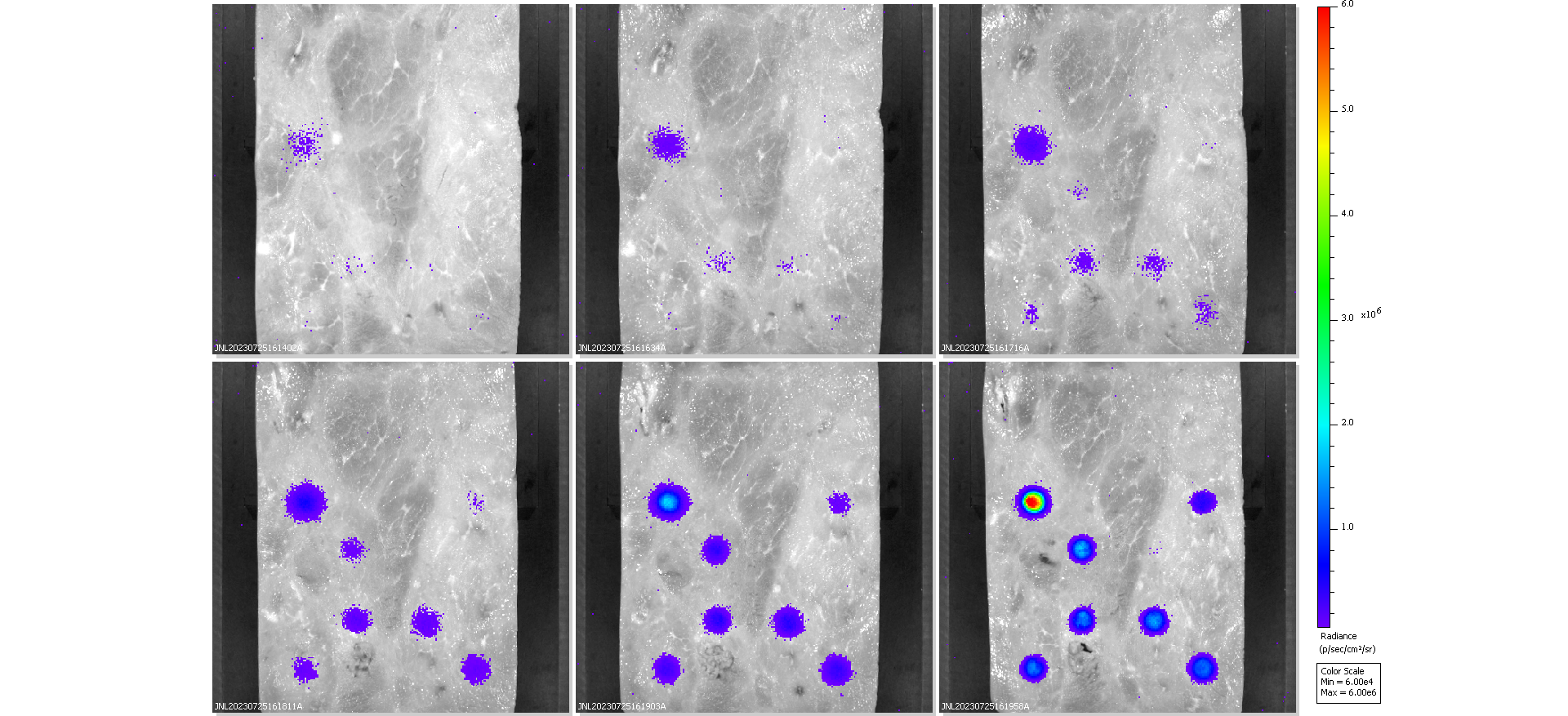

Supplement: Supplement 3 — • Supplementary Data 2: Raw and analyzed source data for Figures 2b, 2d, S3a–c, S4a–b, S6, 3b, 3d, S7c, s9e, 5c–f, S10–S12, 6c–d; source images for westerns blots (Figures s5b, s9b), and IVIS data (Figures 4b, 4d, 6, S8, S9e, S10, S12 (.Zip) [file media-3.zip › Supplementary Data 2/rawImages/s12/s12-rep1-allham.png]

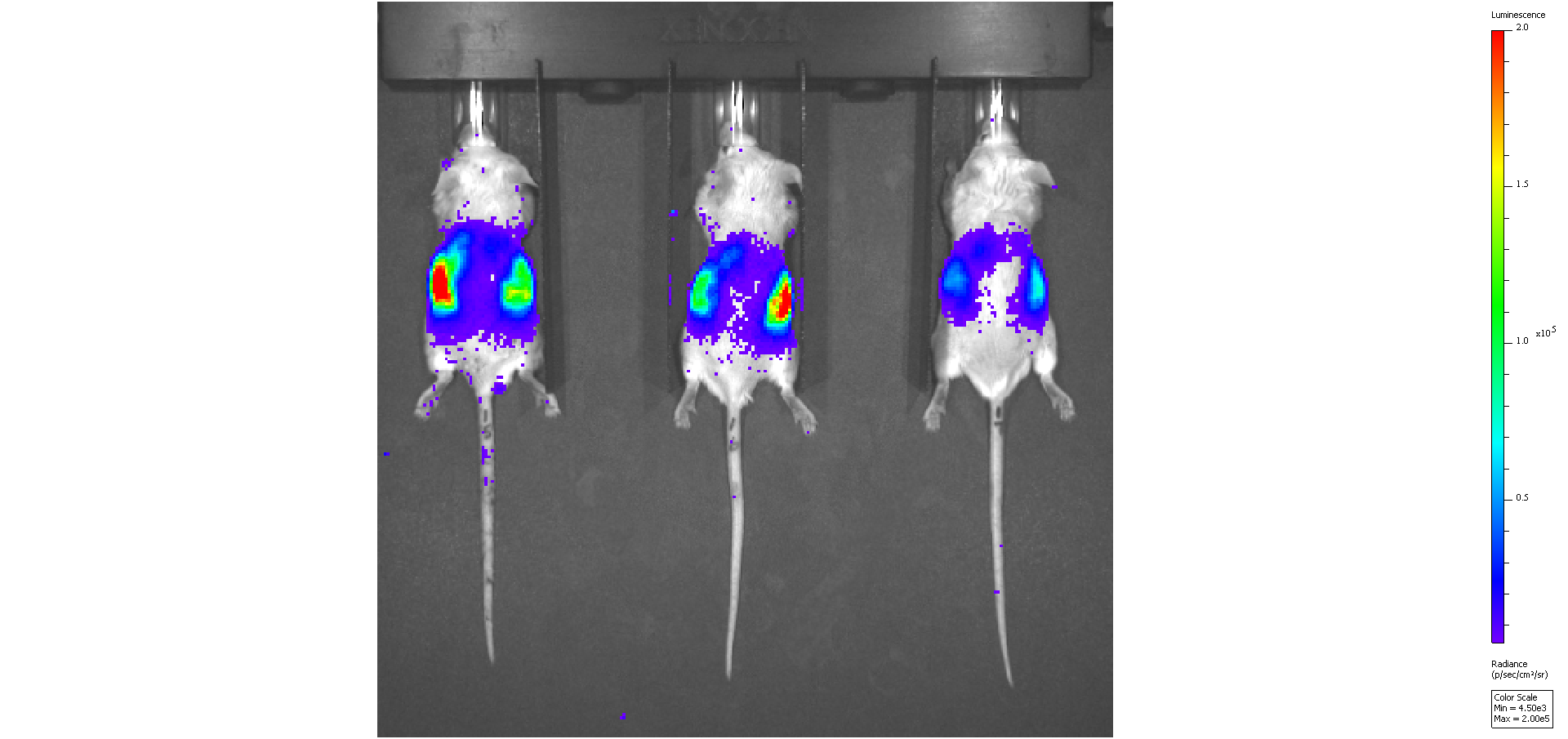

Supplement: Supplement 3 — • Supplementary Data 2: Raw and analyzed source data for Figures 2b, 2d, S3a–c, S4a–b, S6, 3b, 3d, S7c, s9e, 5c–f, S10–S12, 6c–d; source images for westerns blots (Figures s5b, s9b), and IVIS data (Figures 4b, 4d, 6, S8, S9e, S10, S12 (.Zip) [file media-3.zip › Supplementary Data 2/rawImages/6/Group3_L3_V2_S2/15-16-17_2m.png]

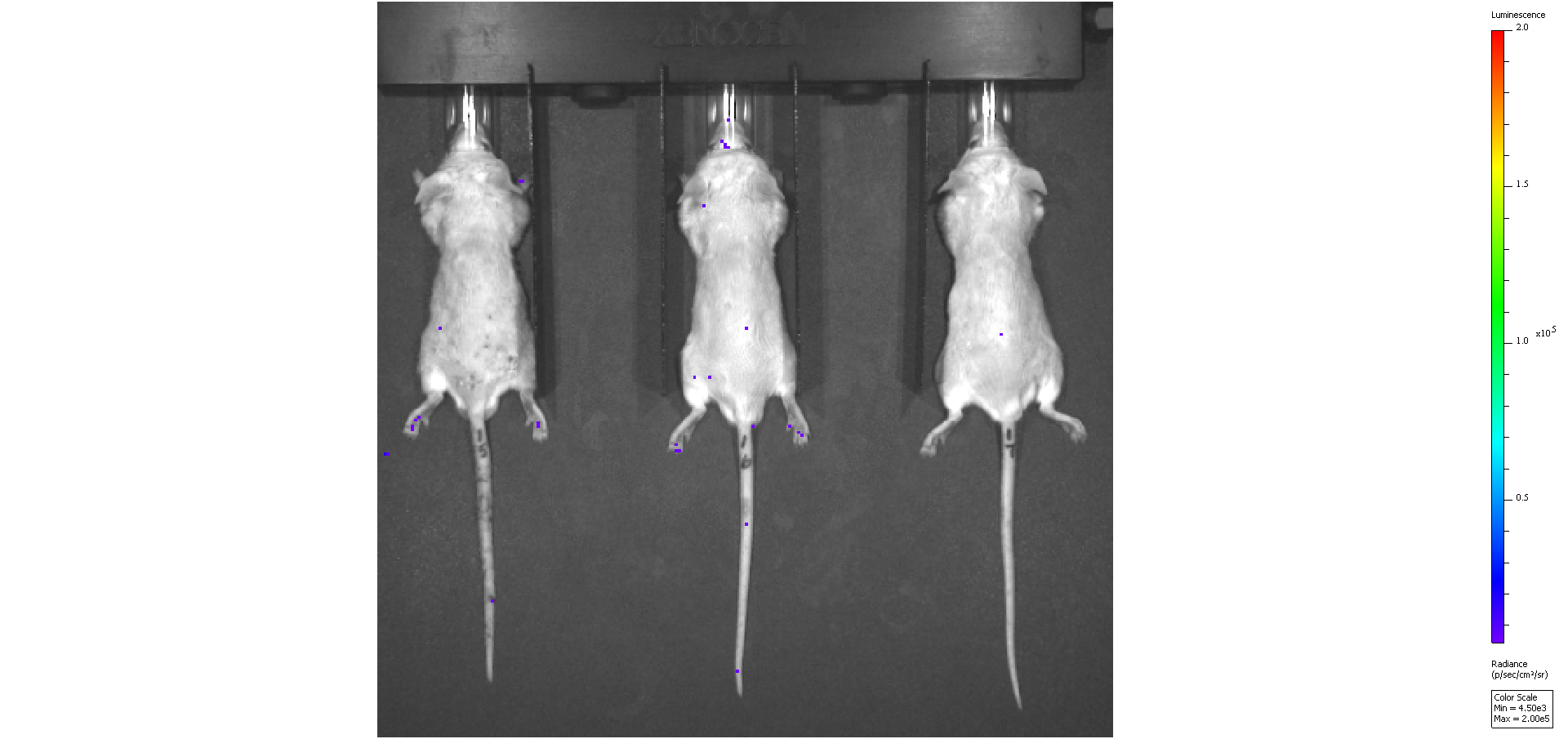

Supplement: Supplement 3 — • Supplementary Data 2: Raw and analyzed source data for Figures 2b, 2d, S3a–c, S4a–b, S6, 3b, 3d, S7c, s9e, 5c–f, S10–S12, 6c–d; source images for westerns blots (Figures s5b, s9b), and IVIS data (Figures 4b, 4d, 6, S8, S9e, S10, S12 (.Zip) [file media-3.zip › Supplementary Data 2/rawImages/6/Group3_L3_V2_S2/15-16-17_Baseline.png]

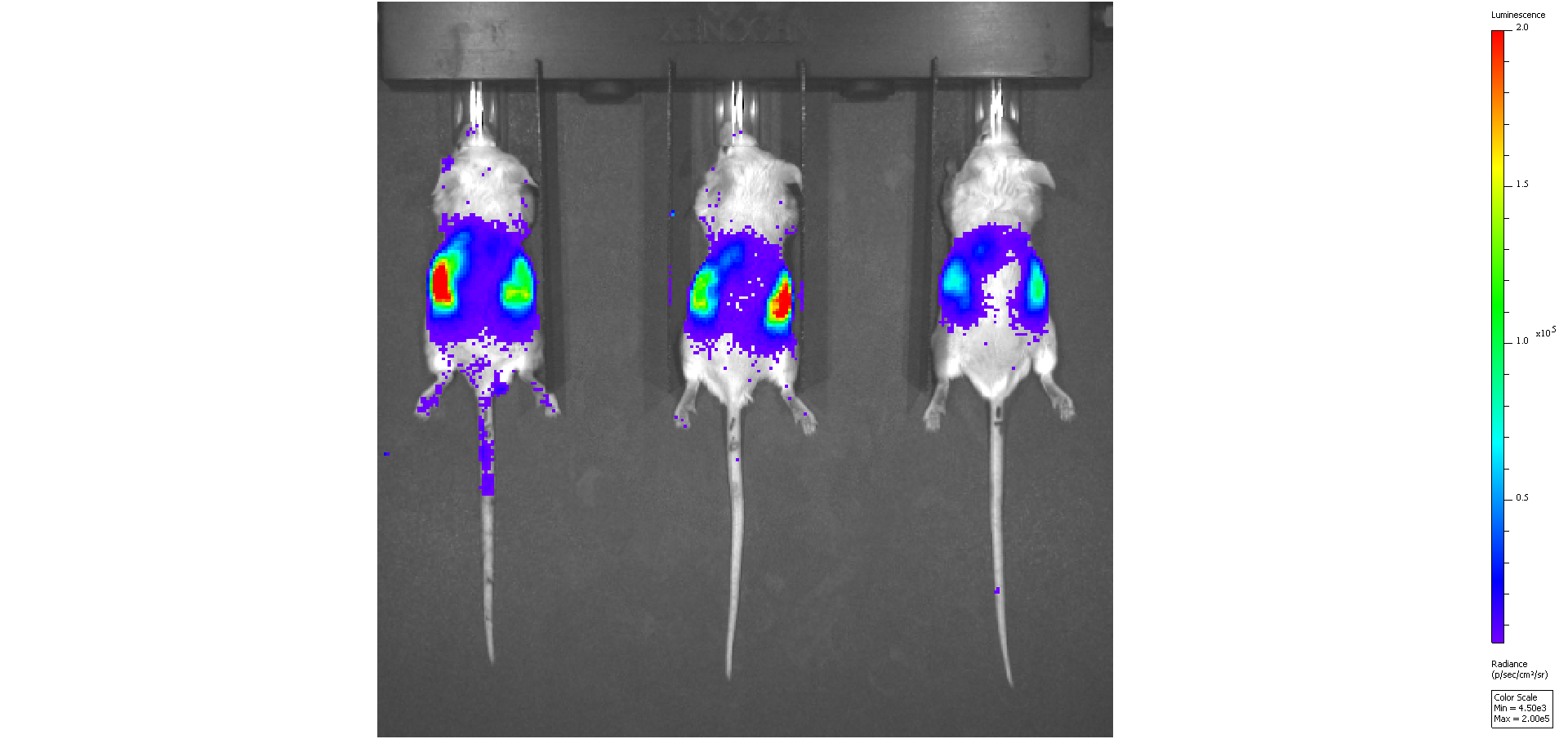

Supplement: Supplement 3 — • Supplementary Data 2: Raw and analyzed source data for Figures 2b, 2d, S3a–c, S4a–b, S6, 3b, 3d, S7c, s9e, 5c–f, S10–S12, 6c–d; source images for westerns blots (Figures s5b, s9b), and IVIS data (Figures 4b, 4d, 6, S8, S9e, S10, S12 (.Zip) [file media-3.zip › Supplementary Data 2/rawImages/6/Group3_L3_V2_S2/15-16-17_7m.png]

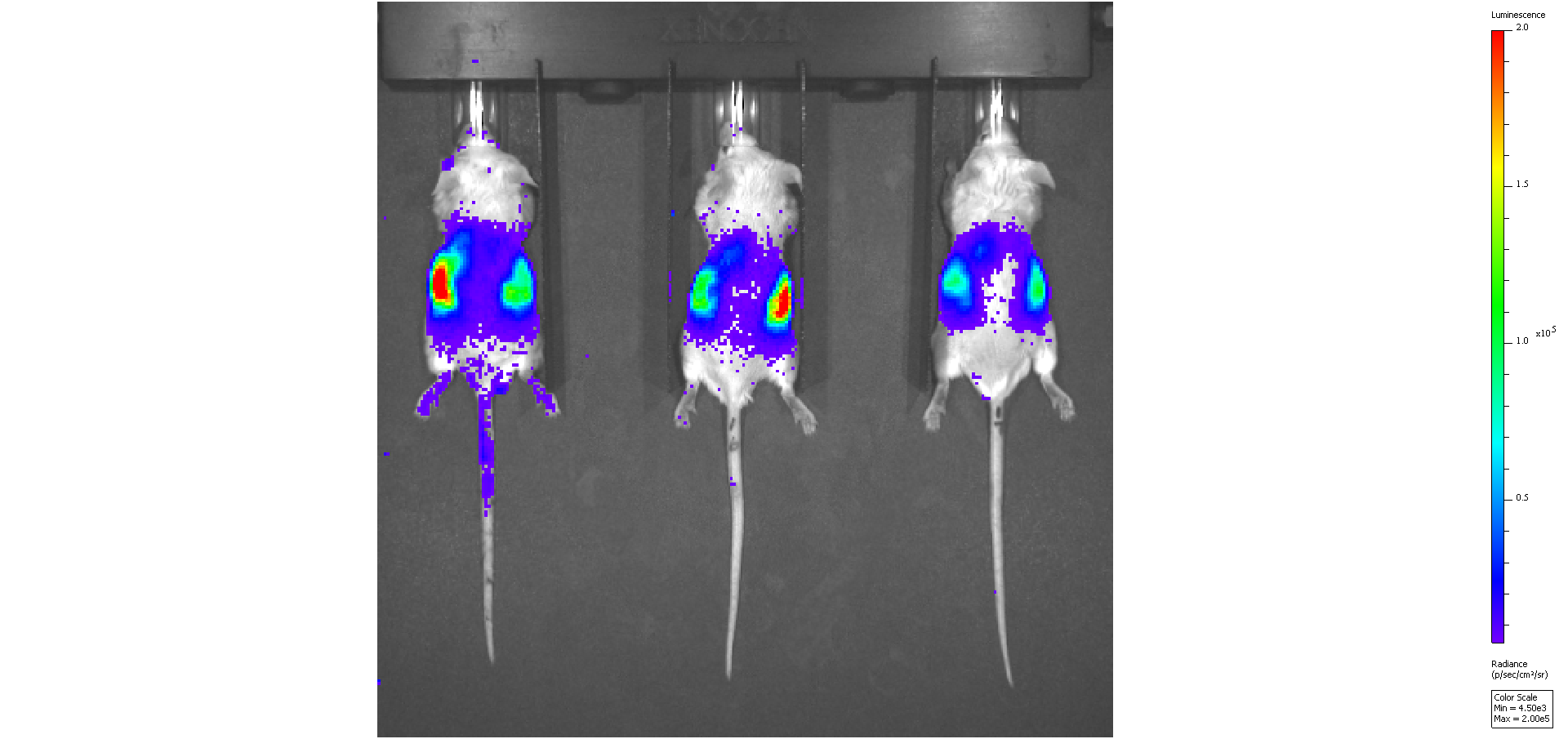

Supplement: Supplement 3 — • Supplementary Data 2: Raw and analyzed source data for Figures 2b, 2d, S3a–c, S4a–b, S6, 3b, 3d, S7c, s9e, 5c–f, S10–S12, 6c–d; source images for westerns blots (Figures s5b, s9b), and IVIS data (Figures 4b, 4d, 6, S8, S9e, S10, S12 (.Zip) [file media-3.zip › Supplementary Data 2/rawImages/6/Group3_L3_V2_S2/15-16-17_12m.png]

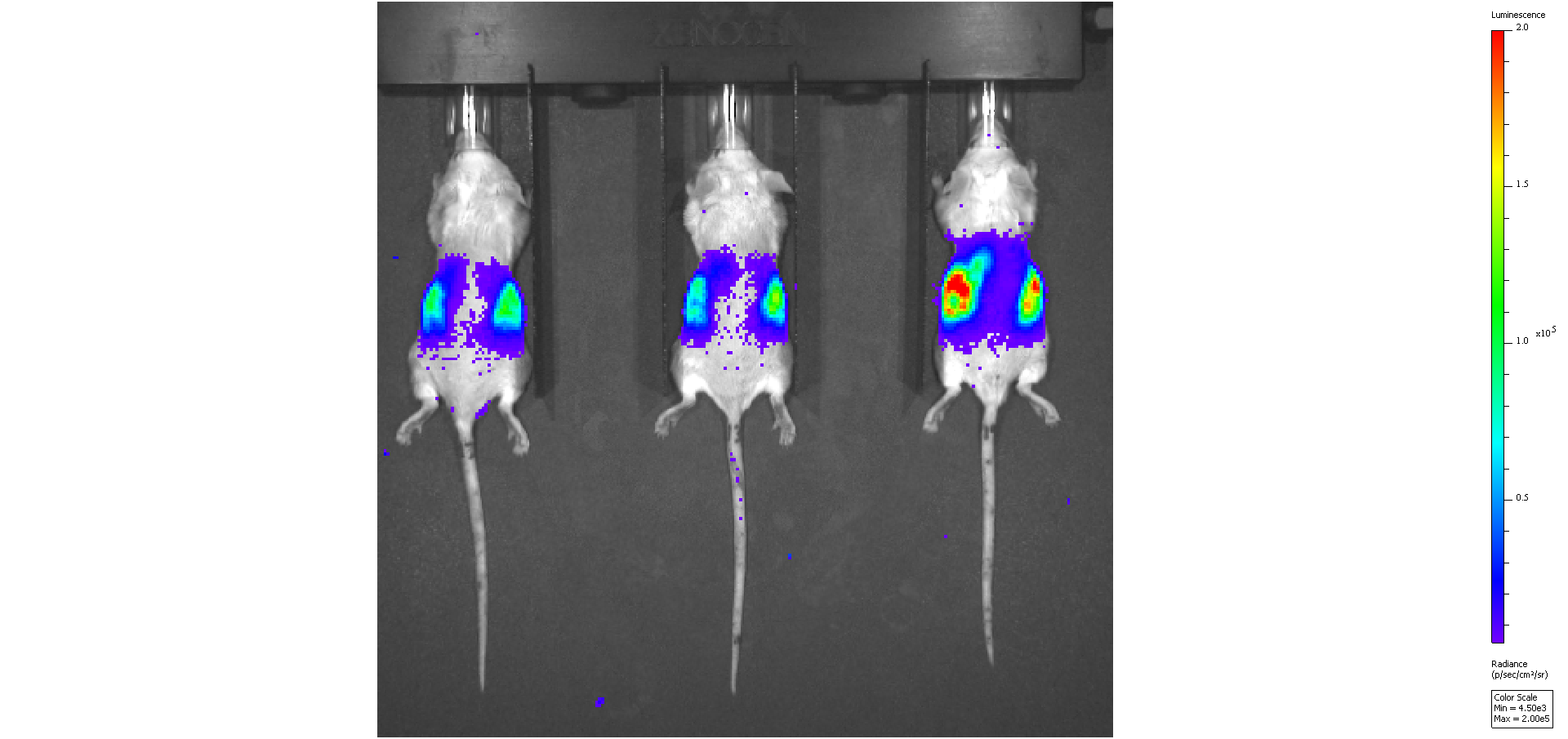

Supplement: Supplement 3 — • Supplementary Data 2: Raw and analyzed source data for Figures 2b, 2d, S3a–c, S4a–b, S6, 3b, 3d, S7c, s9e, 5c–f, S10–S12, 6c–d; source images for westerns blots (Figures s5b, s9b), and IVIS data (Figures 4b, 4d, 6, S8, S9e, S10, S12 (.Zip) [file media-3.zip › Supplementary Data 2/rawImages/6/Group2_V1_V3_L2/12-13-14_2m.png]

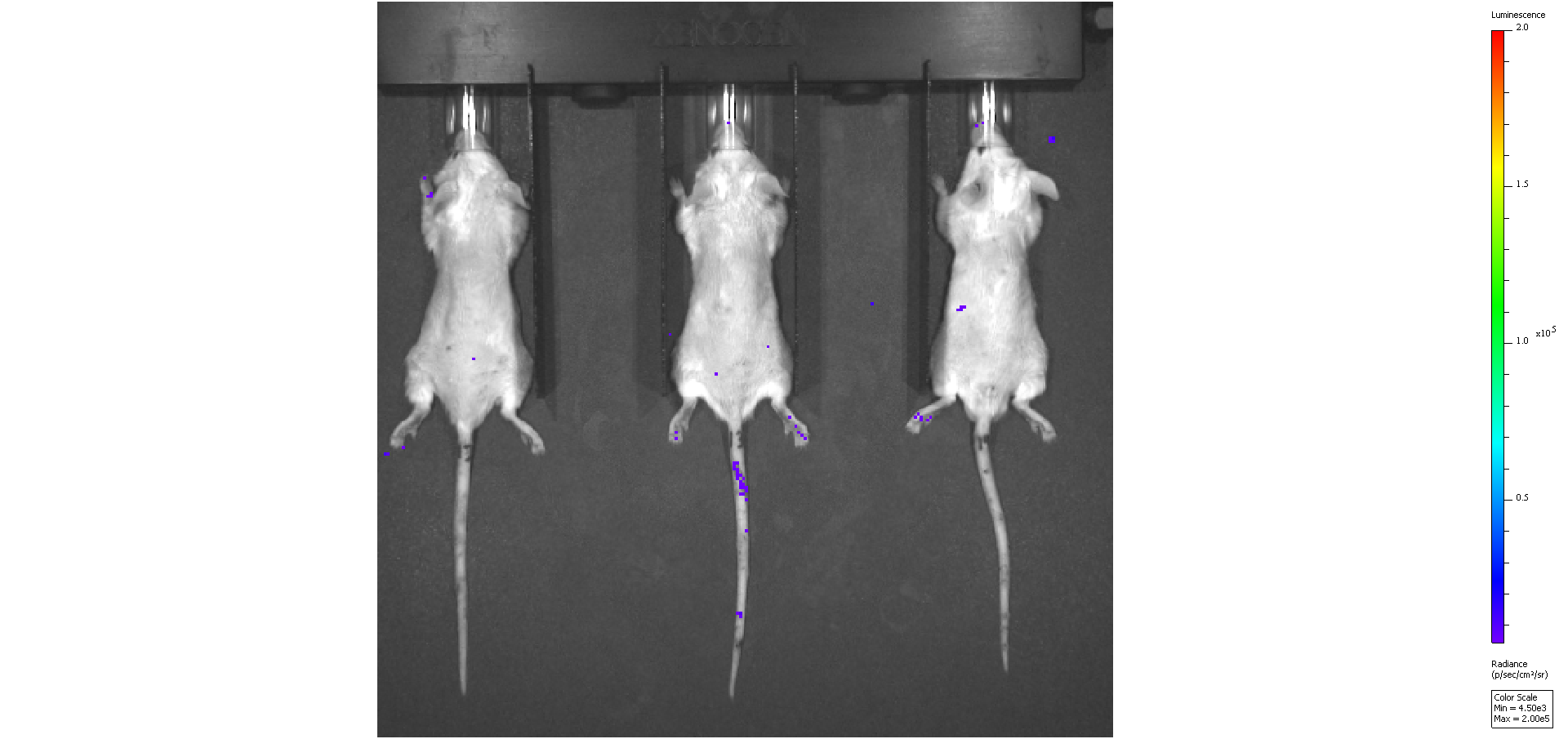

Supplement: Supplement 3 — • Supplementary Data 2: Raw and analyzed source data for Figures 2b, 2d, S3a–c, S4a–b, S6, 3b, 3d, S7c, s9e, 5c–f, S10–S12, 6c–d; source images for westerns blots (Figures s5b, s9b), and IVIS data (Figures 4b, 4d, 6, S8, S9e, S10, S12 (.Zip) [file media-3.zip › Supplementary Data 2/rawImages/6/Group2_V1_V3_L2/12-13-14_Baseline.png]

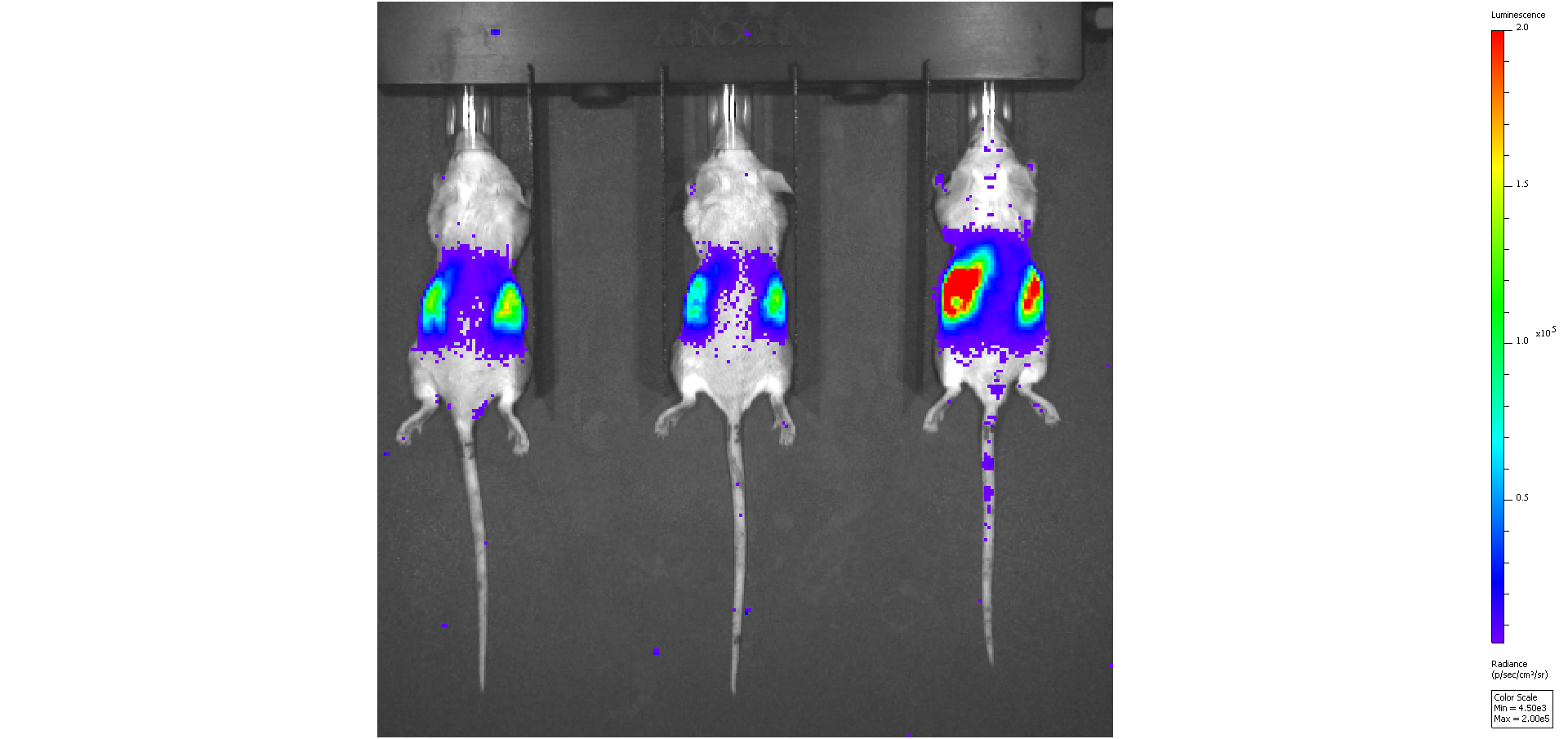

Supplement: Supplement 3 — • Supplementary Data 2: Raw and analyzed source data for Figures 2b, 2d, S3a–c, S4a–b, S6, 3b, 3d, S7c, s9e, 5c–f, S10–S12, 6c–d; source images for westerns blots (Figures s5b, s9b), and IVIS data (Figures 4b, 4d, 6, S8, S9e, S10, S12 (.Zip) [file media-3.zip › Supplementary Data 2/rawImages/6/Group2_V1_V3_L2/12-13-14_7m.png]

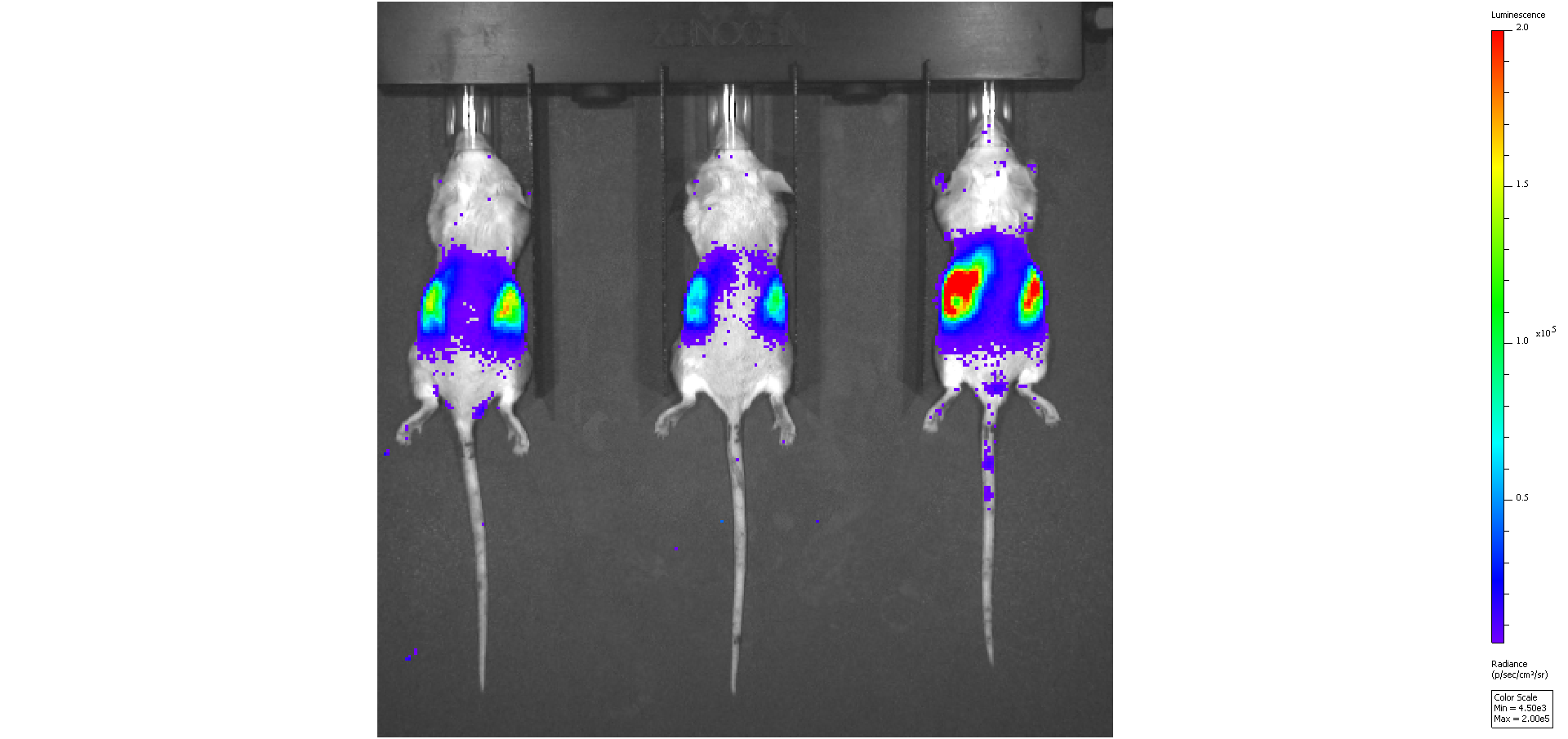

Supplement: Supplement 3 — • Supplementary Data 2: Raw and analyzed source data for Figures 2b, 2d, S3a–c, S4a–b, S6, 3b, 3d, S7c, s9e, 5c–f, S10–S12, 6c–d; source images for westerns blots (Figures s5b, s9b), and IVIS data (Figures 4b, 4d, 6, S8, S9e, S10, S12 (.Zip) [file media-3.zip › Supplementary Data 2/rawImages/6/Group2_V1_V3_L2/12-13-14_12m.png]

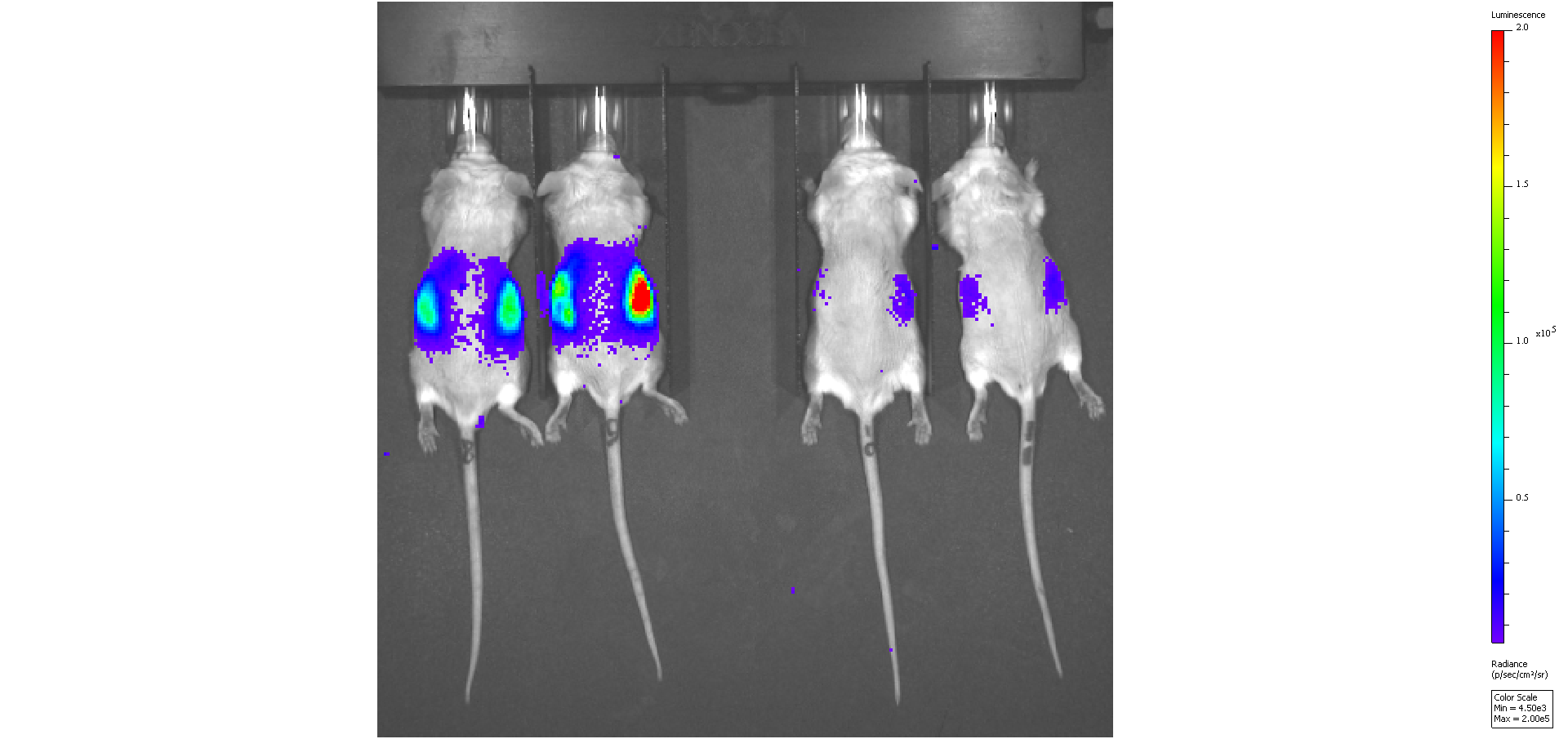

Supplement: Supplement 3 — • Supplementary Data 2: Raw and analyzed source data for Figures 2b, 2d, S3a–c, S4a–b, S6, 3b, 3d, S7c, s9e, 5c–f, S10–S12, 6c–d; source images for westerns blots (Figures s5b, s9b), and IVIS data (Figures 4b, 4d, 6, S8, S9e, S10, S12 (.Zip) [file media-3.zip › Supplementary Data 2/rawImages/6/Group1_S1_L1/8-9-10-11_12m.png]

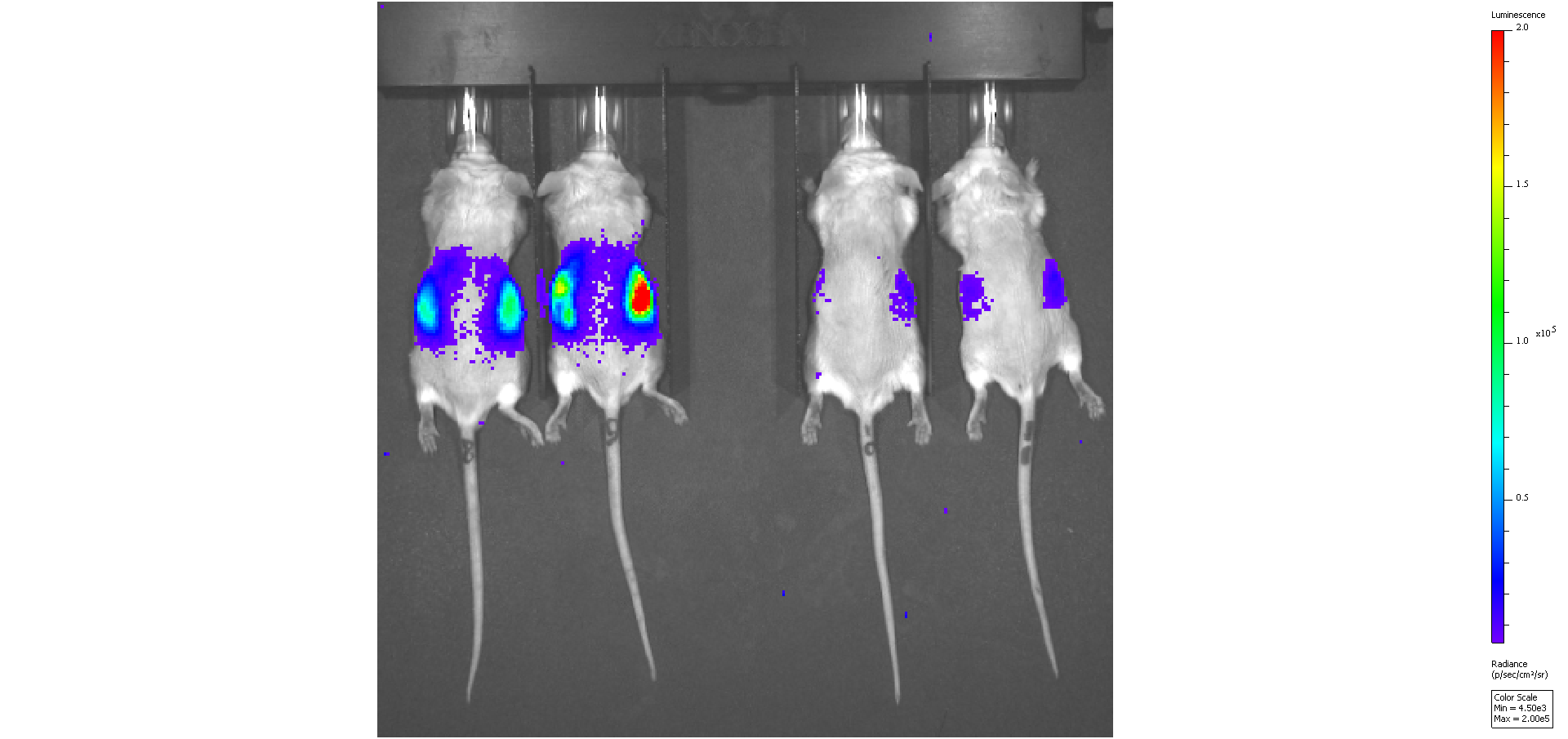

Supplement: Supplement 3 — • Supplementary Data 2: Raw and analyzed source data for Figures 2b, 2d, S3a–c, S4a–b, S6, 3b, 3d, S7c, s9e, 5c–f, S10–S12, 6c–d; source images for westerns blots (Figures s5b, s9b), and IVIS data (Figures 4b, 4d, 6, S8, S9e, S10, S12 (.Zip) [file media-3.zip › Supplementary Data 2/rawImages/6/Group1_S1_L1/8-9-10-11_7m.png]

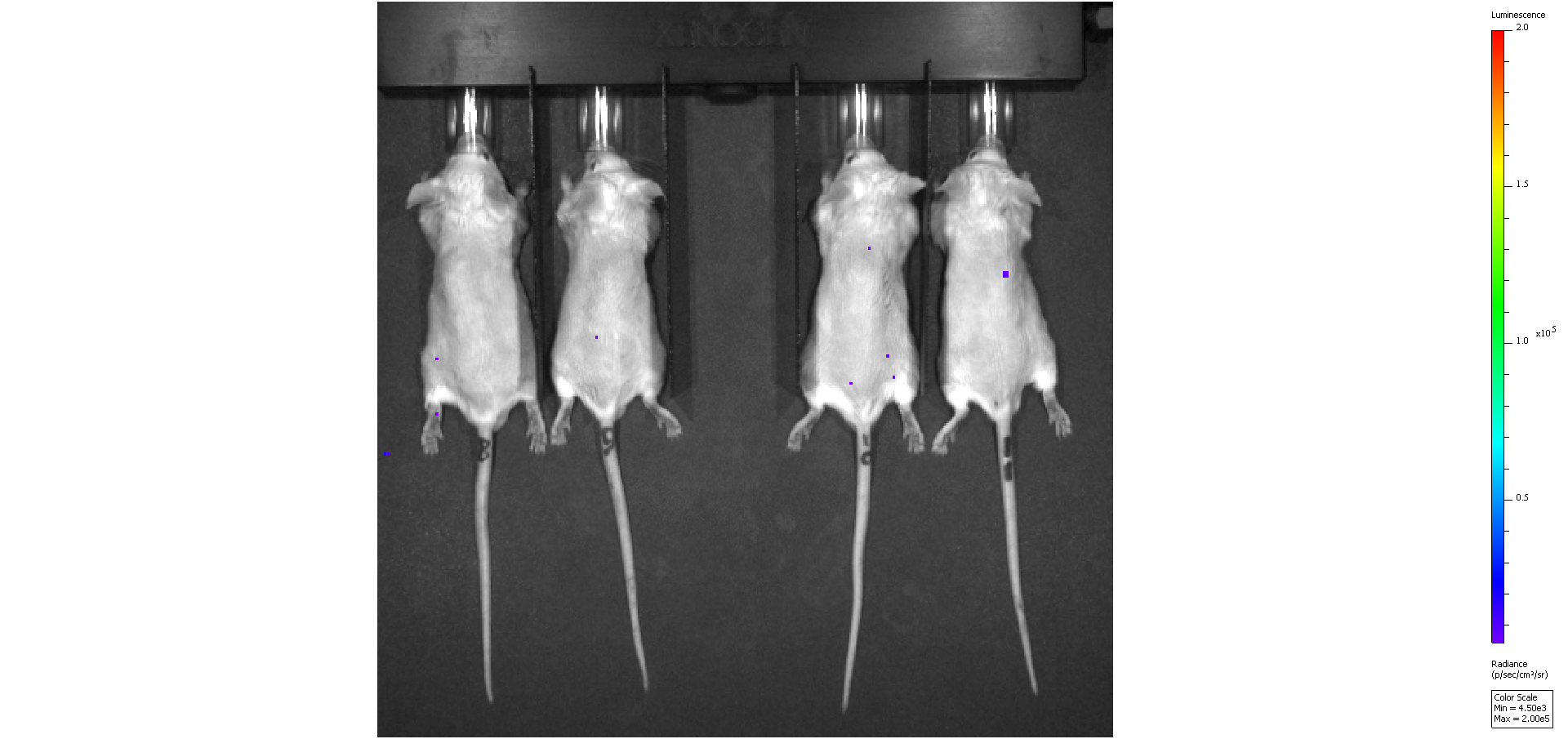

Supplement: Supplement 3 — • Supplementary Data 2: Raw and analyzed source data for Figures 2b, 2d, S3a–c, S4a–b, S6, 3b, 3d, S7c, s9e, 5c–f, S10–S12, 6c–d; source images for westerns blots (Figures s5b, s9b), and IVIS data (Figures 4b, 4d, 6, S8, S9e, S10, S12 (.Zip) [file media-3.zip › Supplementary Data 2/rawImages/6/Group1_S1_L1/8-9-10-11_Baseline.png]

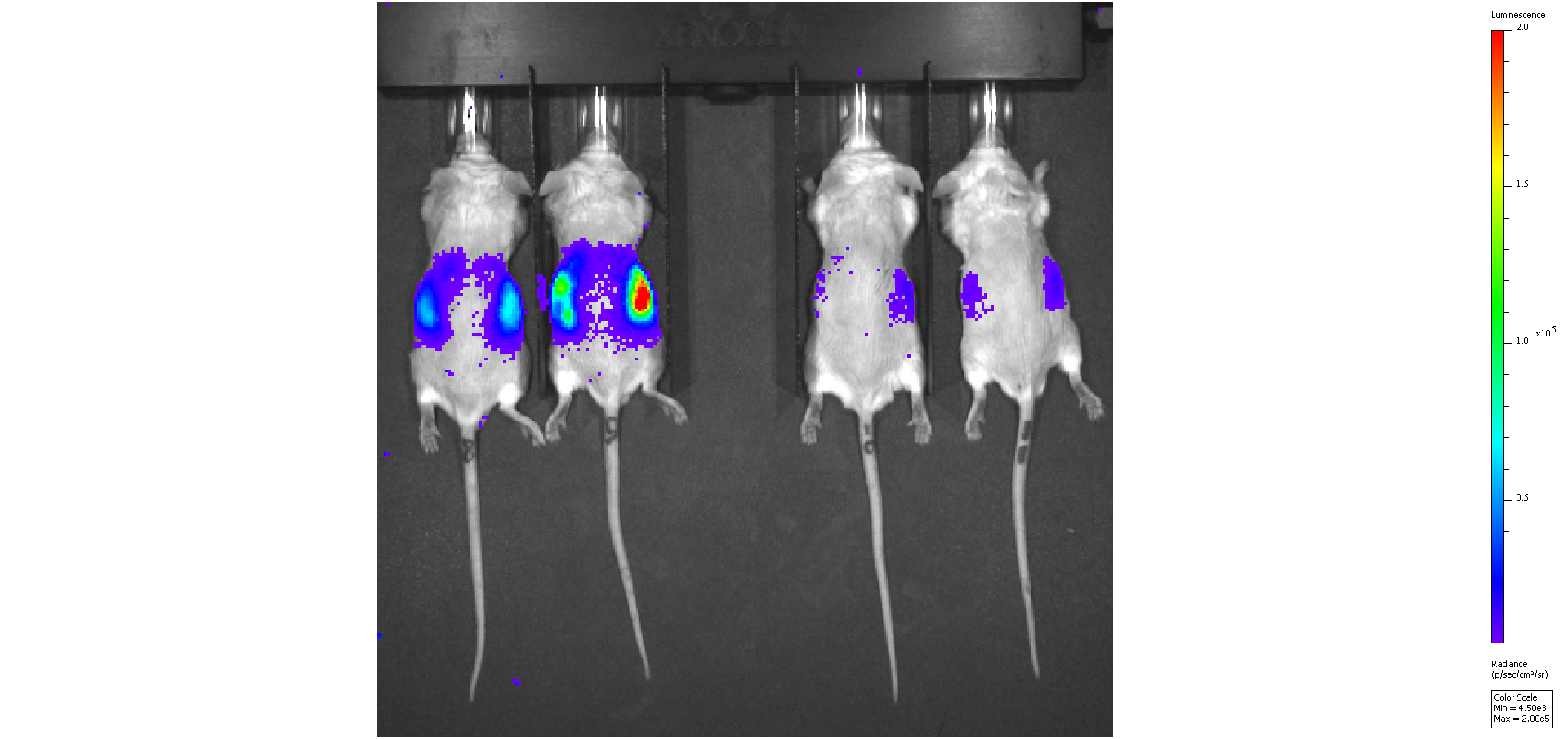

Supplement: Supplement 3 — • Supplementary Data 2: Raw and analyzed source data for Figures 2b, 2d, S3a–c, S4a–b, S6, 3b, 3d, S7c, s9e, 5c–f, S10–S12, 6c–d; source images for westerns blots (Figures s5b, s9b), and IVIS data (Figures 4b, 4d, 6, S8, S9e, S10, S12 (.Zip) [file media-3.zip › Supplementary Data 2/rawImages/6/Group1_S1_L1/8-9-10-11_2m.png]
